# Targeting multiple hallmarks of mammalian aging with combinations of interventions

**DOI:** 10.18632/aging.206078

**Published:** 2024-08-18

**Authors:** Alexander Y. Panchin, Anna Ogmen, Artem S. Blagodatski, Anastasia Egorova, Mikhail Batin, Timofey Glinin

**Affiliations:** 1 Sector of Molecular Evolution, Institute for Information Transmission Problems, Russian Academy of Sciences, Moscow 127051, Russia; 2 Open Longevity, Sherman Oaks, CA 91403, USA; 3 Department of Molecular Biology and Genetics, Bogazici University, Istanbul 34342, Turkey; 4 Institute of Theoretical and Experimental Biophysics, Russian Academy of Sciences, Pushchino 142290, Russia; 5 Department of Surgery, Endocrine Neoplasia Laboratory, University of California, San Francisco, CA 94143, USA

**Keywords:** aging, hallmarks, combination therapy, lifespan and healthspan, mouse, synergistic effect

## Abstract

Aging is currently viewed as a result of multiple biological processes that manifest themselves independently, reinforce each other and in their totality lead to the aged phenotype. Genetic and pharmaceutical approaches targeting specific underlying causes of aging have been used to extend the lifespan and healthspan of model organisms ranging from yeast to mammals. However, most interventions display only a modest benefit. This outcome is to be expected if we consider that even if one aging process is successfully treated, other aging pathways may remain intact. Hence solving the problem of aging may require targeting not one but many of its underlying causes at once. Here we review the challenges and successes of combination therapies aimed at increasing the lifespan of mammals and propose novel directions for their development. We conclude that both additive and synergistic effects on mammalian lifespan can be achieved by combining interventions that target the same or different hallmarks of aging. However, the number of studies in which multiple hallmarks were targeted simultaneously is surprisingly limited. We argue that this approach is as promising as it is understudied.

## INTRODUCTION

Aging is a time-related process that results in health deterioration and an exponential increase in mortality rate [1]. Contemporary geroscience is focused both on investigating the underlying causes of aging and discovering interventions that can prolong animal lifespan. Among mammals, mice are by far the most popular model organism of aging research; however, the attempts to prolong murine lifespan have so far produced only modest results.

The maximum known life extension of mice, resulting from a single intervention, does not exceed 50% (Snell mice with *Pit1* knockout or Ames mice with *Prop1* knockout). Even in these cases the lifespan of mice is much lower than that of similarly sized mammals with negligible senescence such as the naked mole-rat *Heterocephalus glaber* [2], indicating that none of these interventions was sufficient to stop aging. One possible explanation is that even if one underlying cause of aging is countered, the remaining aging processes will still limit the animal’s lifespan. Thus, we propose the hypothesis that combination therapies can be more efficient against aging.

Combination therapy involves two or more interventions or therapeutic agents that can act synergistically, additively or reduce the side effects of one another. This principle has become the gold standard in various fields of medicine including HIV antiviral therapies [3], cancer treatment [4] and overcoming the evolution of drug resistance [5] such as bacterial resistance to antibiotics [6].

Targeting multiple pathways at once can provide synergistic effects that are expected to be greater than the simple sum of independent effects. For example, chemotherapy kills cancer cells leading to proliferation of cancer-targeting T cells. However, some cancers evolve adaptations that suppress this immune response. Checkpoint inhibitors can lift this suppression allowing T cells to be more effective. Several clinical trials have revealed that chemotherapy works better when combined with this form of immunotherapy [7, 8].

Similar examples can be found in the field of aging research. Using the model organism *C. elegans* Chen and Lahav have shown that a ribosomal protein S6 kinase beta deletion allele, *daf-2* loss-of-function allele and their combined effects increase the worm’s lifespan by 20%, 168.8% and 454.4% respectively [9]. Castillo-Quan et al. have shown that a combination of trametinib, rapamycin and lithium increase the longevity of *Drosophila* more than each single intervention or pairs of interventions. These drugs inhibit mitogen-activated protein kinase kinase, mTOR complex 1 and glycogen synthase kinase-3 respectively, thus targeting various components of the nutrient-sensing network [10]. Recently Kaur et al. showed a synergistic effect of cyclically induced expression of Yamanaka factors (*Oct4, Klf4, Sox2, c-Myc*) and senolytic peptide (FOXO4-DRI) for lifespan extension in *Drosophila* [11]. Generally, as aging-related pathologies are typically comorbid, targeting multiple biological processes or their separated nodes may be more effective than targeting a single one.

Another rationale for the use of combination therapies is that two treatments can improve each other’s effects on a single process. For example, senolytics dasatinib and quercetin have different cell-type specificity and are used together as a cocktail to improve senescent cell clearance [12]. A third rationale is to use a treatment that reduces the negative side effects of another. Early experiments on lifespan extension via increased telomerase reverse transcriptase (TERT) activity involved mice with cancer resistant genetic backgrounds with enhanced expression of the tumor suppressors p53, p16, and p19^ARF^ to counter the supposed oncogenic effect of telomerase [13]. Although further studies did not find evidence of increased cancer rates due to telomerase gene therapy and lifespan extension by telomerase alone was of comparable magnitude in regular mice [14, 15].

Currently, the most comprehensive analysis of synergistic anti-aging interactions is provided by the SynergyAge database [16] which contains the current state of the art collection of data on long-lived and short-lived genetic mutants with over 1800 gene combinations. However, the database does not cover pharmacological and gene therapy interventions which are arguably more relevant for practical human lifespan extending applications.

Here we review existing data on combinations of pharmacological and genetic interventions targeting one or many pathological processes described as the hallmarks of aging in mice [17, 18]. While we also discuss studies performed on other mammals, we focus on mice because they were used in the largest number of longevity intervention studies. There are important limitations for using mice as a model, given their short lifespan and high cancer mortality. Other models such as dogs are gaining increased attention in geroscience [19], however longevity studies in dogs are still relatively few.

Our ultimate goal is to design and propose several combination therapies that can be tested in mice and in the case of their success translated into human trials, providing a proof-of-principle for this approach.

### Methodological considerations

While our fundamental understanding of the underlying causes of aging is still incomplete [20], for the purpose of this review we will consider aging as a complex pathological phenomenon that is characterized by several hallmarks. This is practically useful, because the hallmarks of aging are specifically targetable by known interventions. Up to date the most reliable benchmark for determining the effectiveness of anti-aging interventions is increased lifespan. Thus, our review focuses on lifespan extending interventions; however, we provide a separate analysis for the outcomes of combined therapies on other aging-related conditions.

As of 2023 the following twelve hallmarks of aging (HAs) have been proposed [21]: genomic instability (GI), telomere attrition (TA), epigenetic alterations (EA), loss of proteostasis (LP), disabled macroautophagy (DM), deregulated nutrient-sensing (DNS), mitochondrial dysfunction (MitD), cellular senescence (CS), stem cell exhaustion (SCE), altered intercellular communication (AIC), chronic inflammation (Inf) and dysbiosis (Dys). This is an expansion of the previous list published in 2013 by Lopez-Otin et al. [17].

Some authors suggested that additional hallmarks should be considered such as splicing dysregulation (SD), and altered mechanical properties (AMP), which includes stochastic non-enzymatic modification of long-lived macromolecules [18, 22]. Additionally, specific aging contributors such as thymic involution and immune system decline have been highlighted, which we will include in an additional hallmark called immunaging (IA) [23] (Box 1). These additional hallmarks are added because they appear in the scientific literature, and can also be specifically targeted by anti-aging interventions.

**Box 1. List of hallmarks of aging described up to date.**

Genomic instability GI (+)

Telomere attrition TA (+)

Epigenetic alterations EA (+)

Loss of proteostasis LP (+)

Deregulated nutrient-sensing DNS (+)

Mitochondrial dysfunction MitD (+)

Cellular senescence CS (+)

Stem cell exhaustion SCE (+)

Altered intercellular communication AIC (+)

Disabled macroautophagy DM (+)

Splicing dysregulation SD (−)

Chronic inflammation Inf (+)

Altered mechanical properties AMP (+)

Dysbiosis Dys (−)

Immunaging IA (+)

The hallmarks for which a known targeted life-prolonging therapy exists in mammals, are marked with (+) otherwise with (−).

Interventions designed to target some of the hallmarks of aging have resulted in life extension of various model organisms. Table 1 provides examples of prominent longevity studies that can be linked with a specific aging hallmark. When attributing hallmarks of aging to a given intervention we considered only the primary processes affected without taking into account all possible secondary effects (e.g., the increase of telomerase activity can reduce stem cell exhaustion and the removal of senescent cells can reduce inflammation). Nevertheless, we still assume that one intervention can target multiple hallmarks of aging.

**Table 1:** Monotherapies aimed at different aging hallmarks, most effective in mammals.

**Therapeutic agent**	**Target**	**HA**	**Studied biological effects**	**Median Lifespan extension compared to control**	**Maximum Lifespan extension compared to control**	**Ref.**
**Telomerase AAV gene therapy**	Telomere shortening	TA	Beneficial effects on health and fitness, including insulin sensitivity, osteoporosis, neuromuscular coordination and several molecular biomarkers of aging. Treatment started in 1- and 2-year-old mice	+ 24% (1 y-o.) + 13% (2 y-o.)	+ 13% (1 y-o.) + 20% (2 y-o.)	[14]
**TERT in CMV vector**			Improved glucose tolerance, physical performance, prevention of body mass loss and alopecia. Amelioration of telomere shortening associated with aging and mitochondrial structure deterioration. Treatment started in 18-month-old mice	+ 41%	+ 42%	[15]
**Fisetin:** 500 ppm in feed, started at 84 weeks	Senescent cells	CS	Reduced senescence and improved healthspan Note that according to the Interventions Testing Program (ITP) positive effect on lifespan was not reproduced [116]	+ 17% *	+ 11%^*^	[39]
**Clearance of p16 expressing cells** in transgenic mice, started in middle age			Slowed down tumor progression and age-related deterioration	+ 27–24%	+ 3–9%^*^	[40]
**Rapamycin:** 8mg/kg/day injections or 126 ppm in feed started in midage	mTOR, autophagy	DM, LP, DNS	Improved healthspan and microbiome composition	+ 5% (injection) + 9% (feed)	n.s. (injection) + 2% (feed)^*^	[41]
**Rapamycin:** 42 ppm at 20 months			Impaired glucose tolerance	+ 4–15% ♀ + 9–11% ♂	+ 4–12% ♀ + 5–9% ♂	[42]
**Rapamycin:** 14 mg/kg started at 9 m.o.			Attenuated age-associated decline in spontaneous activity in males but not in females	+ 18% ♀ + 10% ♂	+ 13% ♀ + 16% ♂	[43]
**Rapamycin:** 42 ppm started at 9 m.o.			Decreased frailty and aging phenotype, slowed down reproductive maturation, transcriptome and epigenome resemble young state	+ 26% ♀ + 23% ♂	+ 11% ♀^*^ + 8% ♂^*^	[44]
**Rapamycin:** 42 ppm in mothers diet and early in life until 45 days old			The mice grew slower and remained smaller than controls for their entire lives. Their reproductive age was delayed without affecting offspring numbers. The treatment helped to preserve health as measured by frailty index scores, gait speed, and glucose and insulin tolerance tests, the liver transcriptome and epigenome of treated mice were younger at the completion of treatment	n.s. ♀ + 12% ♂	n.s. ♀ + 11% ♂	[45]
**Rapamycin:** 14 ppm after 600 days			Lifespan increase	+ 13% ♀ (mean) + 9% ♂ (mean)	+ 14% ♀ + 9% ♂	[46]
**Metformin:** 0.1% in feed starting from 12 months	AMPK, glucose levels		Preserved physical and mitochondrial functioning, mimicked CR. Finding not reproduced by ITP [47]	+ 5% ♂ (mean)	+ 4% ♂^*^	[48]
**Methionine restriction**, lifelong	Glutathione metabolism		Decreased body weight and higher glutathione levels in serum	+ 43% (mean)	+ 44%	[49]
**Adipose tissue-specific knockout of an insulin receptor**	Metabolism	DNS	Reduced fat mass, protection against age-related obesity and its subsequent metabolic abnormalities, despite normal food intake	+ 18% (mean)	+ 13.8%^*^	[50]
**Klotho overexpression**	Metabolism/ insulin pathway	DNS, AIC	Increased insulin resistance, inhibited insulin/IGF1 signaling	+ 19% ♀ + 31% ♂	+ 7–11% ♀^*^ + 13–26% ♂^*^	[51]
**Alpha-ketoglutarate:** 2% CaAKG in feed	Chronic inflammation	DNS, Inf	Decreased chronic inflammation	+ 11–17% ♀ + 10–13% ♂	+ 8–20% ♀ n.s. ♂	[52]
**0.3–0.5 decrease core body temperature** in transgenic mice	Overexpression the UCP2 in hypocretin neurons, Metabolism	N.A.	Elevated hypothalamic temperature, increased energy efficiency by same caloric intake	+ 20% ♀ + 12% ♂	+ 4%♀ + 14%♂	[53]
**Calorie restriction:** 25–65% of calorie restriction	Metabolism	DNS, LP	Lower weight, less tumor formation and improved T-cells proliferation	+ 20–65%	+ 14–51%	[54]
**IGF-1R +/− knockout mice**	GH-IGF pathway	DNS	Lack of dwarfism, normal energy metabolism and nutrient uptake, normal physical activity, fertility and reproduction	+ 33% ♀ n.s. ♂	+ 20% ♀^*^	[55]
**VEGF activation** by AAV-mediated gene therapy	VEGF signaling	AIC, Inf	Slowed down aging-related pathologies, i.e. inflammaging	+ 39% ♀ + 49% ♂	+ 34% ♀^*^ + 37% ♂^*^	[56]
**Becn1F121A/F121A mutation in beclin 1 that decreases its interaction with the negative regulator, Bcl-2** (increase in autophagy efficiency)	Autophagy	AIC, DM	Improved aging-related pathologies	+ 12%	+ 8%	[57]
**Angiotensin type 1 receptor knockout**	RAAS system	AIC	Slowed down aging of vascular system and protected organs from oxidative damage	+ 24%^*^	+ 26%	[58]
**Ames mice, Prop1 mutation**	Hypothalamus		Long-term reduction of electrical synapse strength between the inhibitory neurons of the thalamic reticular nucleus	+ 85% ♀ + 62% ♂	+ 49%	[59]
**Snell mice, Pit1 mutation**			Growth hormone deficiency	+ 51% ♀ + 29% ♂	+ 24% ♀ + 27% ♂	[60]
**Dwarf mice, mutation in GHRHR** (GH-releasing hormone receptor gene)			Delays in age-dependent collagen cross-linking and in six age-sensitive indices of immune system status	+ 42%	+ 15%^*^	[61]
**p66shc mutant mice**			Induced stress resistance	+ 30%	+ 29%^*^	[62]
**Bone marrow transplantation**	Hematopoietic stem cells	SCE	High rate of bone marrow chimerism	N.A.	+ 28%	[63]
			Reductions of frailty measures, increases in food intake and body weight of aged recipients	+ 12%	+ 5%^*^	[64]
**Sirt6 overexpression**	Histone acetylation	EA, GI	Lower serum levels of insulin-like growth factor 1 (IGF1), higher levels of IGF-binding protein 1 and altered phosphorylation levels of major components of IGF1 signaling	n.s. ♀ + 10–15% ♂	n.s. ♀ + 13–16% ♂	[65]
			Preservation of hepatic glucose output and glucose homeostasis through an improvement in the utilization of two major gluconeogenic precursors, lactate and glycerol by means of increasing hepatic gluconeogenic gene expression, de novo NAD+ synthesis, and systemically enhancing glycerol release from adipose tissue.	+ 15% ♀ + 27% ♂	+ 15% ♀ + 11% ♂	[66]
**Transgenic mutant Klf1**	Hematopoietic stem cells	EA, Inf	Marked delay in age-related physical performance decline and disease progression as evidenced by physiological and pathological examinations, antitumor immune enhancement in response to tumor cell administration	+ 9% ♂	+ 12% ♂	[67]
**NF-kB inhibition** by gene therapy/knockout in hypothalamus	Hypothalamus inflammaging	Inf	IKKβ ablation in microglia prevents the increase of microglial cells over aging, prevents aging from inducing TNF-α expression not only in microglia but in neighboring cells, improving aging-related muscle weakness and tail collagen cross-linking	+ 23% ♂	+ 20% ♂	[68]
**Atg5 transgenic mice**	Autophagy	LP, DM	Leanness, increased insulin sensitivity and improved motor function. Fibroblasts cultured from Atg5 transgenic mice are more tolerant to oxidative damage and cell death induced by oxidative stress	+ 17%	+ 15%^*^	[69]
**mclk1+/− mice**	mclk1 gene	MitD,GI	Protection from oxidative stress and damage to DNA, reduced stress resistance	+ 15–31%	+ 10–30%^*^	[70]
**FGF21**	Thymus involution, IGF-1	DNS, IA	Protects against age-related thymic involution with an increase in earliest thymocyte progenitors and cortical thymic epithelial cells65, increasing perithymic brown adipose tissue, and elevating thymic T-cell export and naïve T-cell frequencies in old mice. Extends lifespan in mice without reducing food intake or affecting markers of NAD+ metabolism or AMP kinase and mTOR signaling. Acts primarily by blunting the growth hormone/insulin-like growth factor-1 signaling pathway in liver	+ 40% ♀ + 30% ♂	+ 16%*	[71]
**Sirt1**	Brain-specific SIRT1 overexpression	EA	Enhanced neural activity specifically in the dorsomedial and lateral hypothalamic nuclei through increased orexin type 2 receptor (Ox2r) expression	+ 16% ♀ + 9% ♂	+ 6% ♀* + 4% ♂*	[72]
**Macrophage inhibitory factor knockout mice**	Immune system	IA, Inf	Lifespan extension in response to CR, and under standard conditions. Protection against lethal hemangiosarcoma. Enhanced mortality due to disseminated amyloid, an age-related inflammatory syndrome	+ 16%	+ 20%^*^	[73]
**FOXM1 induction**	Cell proliferation	CS, SCE	Mitigation of several cellular aging hallmarks, significantly extending the lifespan of both normal and progeroid aging animals.	+ 26,8%	N.A.	[74]
**Acarbose**	Inhibits alpha glucosidase in the gut	DNS, AMP	Reduced lung tumors in males, diminished liver degeneration in both sexes and glomerulosclerosis in females, reduced blood glucose responses to refeeding in males, and improved rotarod performance in aging females, but not in males. Changed the gut microbiome composition. (ITP study)	+ 2–5% ♀ + 6–22% ♂	+ 3–10% ♀ + 8–12% ♂	[47] [75] [76] [77]
**Spermidine**	Increased autophagy	DM	Enhanced cardiac autophagy, mitophagy and mitochondrial respiration, improved the mechano-elastical properties of cardiomyocytes *in vivo*, coinciding with increased titin phosphorylation and suppressed subclinical inflammation. Lifespan extension not reproduced in rats [78]	+ 10%	+ 5–8%^*^	[78] [79]
**Taurine**	Reduces Hba1c	DNS	Improved functioning of bone, muscle, pancreas, brain, fat, gut, and immune system	+ 10–12%	+ 18–25%	[80]
**Glycine**	Mimics low-methionine diet		Decreased probability of end-of-life carcinogenesis (ITP study)	+ 4% ♀ + 6% ♂	+ 2% ♀ + 2% ♂	[81]
**Follistatin gene therapy**	Increases skeletal muscle mass by neutralizing the effects of various TGF-β ligands involved in muscle fiber breakdown	AIC, MitD	Hair and weight loss prevention, improved activity and motor coordination, improved mitochondrial integrity in muscles, increased glucose tolerance	+ 32,5%	+ 31%^*^	[15]

Abbreviations: GI: genomic instability; TA: telomere attrition; EA: epigenetic alterations; LP: loss of proteostasis; DNS: deregulated nutrient-sensing; Mitd: mitochondrial dysfunction; CS: cellular senescence; SCE: stem cell exhaustion; AIC: altered intercellular communication; DM: disabled macroautophagy; Inf: inflammation; IA: immunaging; AMP: altered mechanical properties. Lifespan extension values deduced from provided mortality curves are marked with (^*^). N.A. is not applicable; n.s. is not statistically significant (mentioned as in article). One important limitation of this approach is that sometimes the attribution of hallmarks to an intervention is based on incomplete knowledge. When possible, we tried to rely on the existing consensus about the primary effects of the intervention, however in some instances we had to rely on the molecular mechanisms suggested by the authors that tested the intervention, even though the mechanism was not definitively proven or widely accepted.

We were able to identify at least one monotherapy that extends the lifespan of normal non-progeroid model mammals for each hallmark of aging except splicing dysregulation and dysbiosis. Although there are no known drugs that extend lifespan by selectively removing modified long-lived macromolecules such as glycated collagen which cause altered mechanical properties of tissues, we speculate that this hallmark of aging can be improved by drugs that lower blood glucose levels such as acarbose or metformin. For example, patients with diabetes that take metformin have lower levels of glycated hemoglobin [24]. It is possible that the drug protects other proteins as well. For dysbiosis we identified one study that found a small increase in lifespan for germ-free mice kept in sterile conditions from birth when compared to mice reared in more conventional settings. This benefit was not present if both groups of mice were food-restricted [25]. Another study found that fecal transplants from wild-type mice increased the lifespan in two progeroid mouse models [26]. There is also limited evidence for lifespan promoting effects of certain probiotics [27].

The design of combined anti-aging therapies should take into account the possible adverse effects of suggested interventions. For example, rapamycin is known for its immunosuppressive properties. Patients taking therapeutic doses of rapamycin have experienced stomatitis, impaired wound healing, thrombocytopenia, and increased levels of serum triglycerides and cholesterol [28]. Doses used to extend the lifespan in mice do not usually result in these side effects; however, impaired glucose homeostasis, gonadal atrophy, and increased incidence of cataracts have been reported [29]. Metformin use can lead to vitamin B12 deficiency, gastrointestinal symptoms and other health problems [30]. Dasatinib use for cancer treatment is linked to pulmonary arterial hypertension and pleural effusion, platelet dysfunction, and a number of gastrointestinal side effects including intestinal bleeding, although the use of dasatinib as a senolytic may involve lower and thus safer dosages of the drug [31]. The loss of function mutations in PROP1 and PIT1 genes of long-lived Ames and Snell mice cause delayed growth and dwarfism. The loss of function of these genes in humans causes severe developmental abnormalities [32, 33] and appear not to increase lifespan [34]. Finally, most gene therapies result in a concomitant burden on the liver and immune system [35, 36] and in some cases, the use of certain vectors is limited due to insertional mutagenesis [37, 38].

The existence of adverse effects for potential anti-aging treatments is especially relevant for healthy individuals, because of the increased risk to benefit ratio. Certain adverse effects might be more acceptable in standard pathology treatment because they are less severe than the disease itself, although we should keep in mind that the adverse effects of aging include increased risk of cancer, cardiovascular diseases, chronic obstructive pulmonary disease, cataracts, severe disability and death. Nevertheless, we must admit that most interventions from Table 1 have not been carefully studied for adverse-effects, which is an important limitation for designing combination therapies. Notably, the adverse effects of some proposed treatments can be potentially offset (or exacerbated) by other components of the combined therapy. For example, immune system depletion can be theoretically offset by bone marrow transplantations and FGF21 inhibition of thymus involution.

## Combination therapy in mammals

Table 2 summarizes studies that examined the effects of combination therapies including both genetic and pharmaceutical interventions on normally aging mammals and led to additive or synergistic increases in lifespan. Table 3 contains known negative results of combination therapies. Table 4 provides examples of combination therapies studied on therapies tested on progeroid and disease mouse models. Progeroid animals are often used as a model for aging because of convenient short initial lifespan, but do not necessarily represent the normal aging process and were thus analyzed separately. Table 5 contains studies of combination therapies that showed various health benefits, but in which lifespan extension was not studied.

**Table 2:** Combined therapies aimed at different aging hallmarks, most effective in rodents.

**Therapy**	**Reasoning**	**Target HA**	**HA over lap**	**Biological effects**	**Effect of combination**	**Separate interventions, median lifespan compared to control**	**Combined interventions, median lifespan compared to control**	**Separate interventions, maximum lifespan compared to control**	**Combined interventions, maximum lifespan compared to control**	**Ref.**
**Metformin + rapamycin:** 1000 ppm and 14 ppm in food from 9 months of age (ITP study)	Both components inhibit different mTOR complexes, and metformin is expected to compensate negative effects of rapamycin	DM, LP, DNS	overlap	Increase in lifespan	Synergistic, except female maximum;	metformin: 0% ♀ + 7% ♂ rapamycin: + 21% ♀ + 13% ♂	+ 23%	metformin: 0% ♀ - 2% ♂ rapamycin: + 11% ♀ + 8% ♂	+ 17% ♀ + 10% ♂	[47] [44]
**Simvastatin + ramipril:** 20 and 5 mg drug/kg bw/d in feed	Combination of drugs effective in decreasing mortality and is used in treatment of aging-related pathologies in humans	DNS		Decreased weight, higher serum glucose levels, increased number of benign tumors and diathesis, which did not affect the lifespan	Synergistic	Simvastatin: 0%^*^ Ramipril: + 3%^*^	+ 9% mean and median	Simvastatin: - 4.1%^*^ Ramipril: + 2%^*^	+ 5%^*^	[89]
**Rapamycin + acarbose started at 9 m.o.:** 14.7 ppm and 1000 ppm in feed (ITP study)	Components target different but overlapping pathways. Rapamycin inhibits mTOR, acarbose reduces glucose levels	DM, LP, DNS, AMP	overlap	Mice gained less weight than controls	Single-component treatment n.a. in this study, comparing with other studies additive in males, neutral in females	n.a.	+ 28% ♀ + 37% ♂	n.a.	+ 21% ♀ + 24% ♂	[90]
**Rapamycin + acarbose stated at 16 m.o.:** 14.7 ppm and 1000 ppm in feed (ITP study)							+ 12% ♀ + 14% ♂		+ 15% ♀ + 18% ♂	
**Glycine + N-Acetylcysteine (GlyNAC):** 1.6 mg/g feed and 1.6 mg/g feed each compound	GlyNAC was shown to improve aging-related dysregulations of multiple biological processes	MitD, GI, DNS, DM	partial overlap	In old mice glutathione levels, markers of oxidative stress, mitochondrial dysfunction, autophagy and genomic damage returned to youthful levels	Synergistic, based on other studies with separated components	n.a.	+ 24%	n.a.	+ 33%^*^	[91]
**Dasatinib + quercetin:** started at 24 m.o., 5 mg/kg and 50 mg/kg; biweekly orally for three days	Targeting senescent cells late in life	CS, Inf	overlap	Senescent cell clearance, improved physical activity	n. a.	n.a.	+ 6%; + 36% median post-treatment lifespan	n.a.	+ 5%	[92]
**p53/TERT + p16/Arf** (transgenic mice)	Tumor-suppressing transgenes to compensate tumor-inducing effect of TERT	GI, TA	Partial overlap	Decreased tumor formation, improved physical function, intestinal barrier function, glucose tolerance and genome integrity, reduced telomere	Synergistic	p53/TERT: n.s. p16/Arf: + 7%	+ 40%	p53/TERT: + 7% p16 + Arf: n.a. in this study	+ 22%^*^	[13]
**p53 + s-ARF (+p15/p16)**	Combination of oncosupressors	GI	overlap	Decreased tumor formation, improved genome stability, lifespan extension	Synergistic	p53: n.s. s-Arf: + 7%	+ 16%	p53: n.s. s-Arf: + 5%	+ 10%^*^	[93]
**Catalase OE in peroxisomes + superoxide dismutase overexpression** (in transgenic mice)	To decrease reactive oxygen species levels	MitD		Lifespan extension	Synergistic	SOD1: n.s. PCAT: + 13%	+ 19%	SOD1: n.s. PCAT: + 6%	0%	[94]
**OCT4 + SOX2 + KLF4** (via viral vectors. Induction of OSK started at 124 weeks old mice)	Epigenetic reprogramming	SCE, EA	overlap	Decreased frailty index and extended lifespan	n.a.	n.a.	+ 109% median post-treatment	n.a.	+ 11%^*^	[95]
**Ames mice (PROP1 deficient) + CR**	Targeting insulin sensitivity	AIC, DNS, DM, LP	overlap	Lifespan extension	Additive	Ames: + 38% CR: + 27%	+ 69%	Ames: + 39% CR: + 35%	+ 61%	[96]
**GHRH KO + CR**	Targeting insulin sensitivity	AIC, DNS, DM LP	overlap	Lifespan extension	Additive	KO: + 45% CR: + 21%	+ 64%^*^	KO: + 30% CR: + 23%	+ 52%^*^	[97]
**Ames mice (PROP1 deficient) + CR**	Targeting insulin senstivity	AIC, DNS, DM, LP	overlap	Further increased insulin sensitivity	Additive	Ames: + 36%^*^ CR: + 26%^*^	+ 68%^*^	Ames: + 39%^*^ CR: + 35%^*^	+ 61%^*^	[98]
**GHR KO + CR**	GH/IGF-1 axis suppression	AIC, DNS, DM, LP	overlap	Lower weight	Additive for female’s maximum lifespan	KO: + 31% CR: + 25%	+ 31%	KO: + 17% CR: + 17%	+ 26%	[99]
**Suppressed GH + calorie restriction** (transgenic rats)	GH/IGF-1 axis suppression	AIC, DNS, DM, LP	overlap	Lower weight, protected rats from age-related kidney pathologies	Additive	Suppressed GH: + 7% CR: + 11%^*^	+ 25%^*^	Suppressed GH: + 10% CR: + 18%	+ 30%	[100]

Abbreviations: GI: genomic instability; TA: telomere attrition; EA: epigenetic alterations; LP: loss of proteostasis; DNS: deregulated nutrient-sensing; Mitd: mitochondrial dysfunction; CS: cellular senescence; SCE: stem cell exhaustion; AIC: altered intercellular communication; DM: disabled macroautophagy; Inf: inflammation; IA: immunaging; AMP: Altered mechanical properties. Lifespan extension values deduced from provided mortality curves are marked with (^*^). N.A. is not applicable; n.s. is not statistically significant (mentioned as in article).

**Table 3:** Combined therapies aimed at different aging hallmarks with no additive or synergistic effect on mammalian lifespan.

**Therapy**	**Reasoning**	**Targeting HA**	**Biological effects**	**Effect of combination**	**Median lifespan compared to control**	**Maximum lifespan compared to control**	**Ref.**
PROP1 deficiency + GHR KO	Combining well-known dwarf mutations that extend lifespan	DNS overlap	Improved glucose and insulin tolerance	Lifespan did not exceed the effect of separate interventions	+ 52% ♀ + 30 % ♂	+ 53% ♀ + 25 % ♂	[129]
CuZnSOD + MnSOD (antioxidant enzymes)	Suppression of oxidative stress	n. a.	n. a.	No effect	0%	0%	[124]
Pycnogenol + quercetin + taxifolin	Suppression of oxidative stress	MitD, Inf	Didn’t extend mice lifespan	N.A.	n.s.	n.s.	[128]
Green tea extract + black tea extract + morin	Suppression of oxidative stress	MitD, Inf	−	N.A.	n.s.	n.s.	[128]
GHR KO + intermittent fasting	Metabolism	DNS, AIC	n.a.	No effect	n.s.	n.s.	[117]
GHR KO + 30% CR (from 2 month)	Metabolism	DNS, AIC	n.a.	No effect	n.s.	n.s.	[99]
GHR KO + CR	Metabolism	DNS, AIC	n.a.	Lifespan did not exceed the effect of separate interventions	n.s.	n.s.	[98]

Abbreviations: GI: genomic instability; TA: telomere attrition; EA: epigenetic alterations; LP: loss of proteostasis; DNS: deregulated nutrient-sensing; MitD: mitochondrial dysfunction; CS: cellular senescence; SCE: stem cell exhaustion; AIC: altered intercellular communication; DM: disabled macroautophagy; Inf: inflammation; IA: immunaging. Lifespan extension values estimated from mortality curves are marked with (^*^). N.A. is not applicable; n.s. is not statistically significant (mentioned as in article).

**Table 4:** Combined therapies aimed at different aging hallmarks, effective in progeroid or disease mammal models.

**Therapy**	**Targeting HA**	**Biological effects**	**Effect of combination**	**Median lifespan compared to control**	**Maximum lifespan compared to control**	**Ref.**
Pravastatin + zoledronate	LP	Reduced progeroid symptoms and extended the lifespan	Synergistic	+ 77%	+ 80%	[136]
ATP + levamisole (TNAP inhibitor) + ARL67156 (eNTPD inhibitor)	AMP	Extended lifespan and improved symptoms in progeria animals by preventing vascular calcification	Synergistic	+ 12%	+ 13%	[134]
CR + fish oil	DNS	Reduced inflammation and increased lifespan	Additive	+ 166%	+ 167%	[138]
Cyclophosphamide + tilorone		Increased lifespan but very slightly compared with cyclophosphamide alone	Additive	+ 73%	N.A.	[139]
OSKM	SC, EA	Reduced progeroid symptoms and extended the lifespan	Synergistic	+ 20%	+ 21%	[137]

Abbreviations: GI: genomic instability; TA: telomere attrition; EA: epigenetic alterations; LP: loss of proteostasis; DNS: deregulated nutrient-sensing; MitD: mitochondrial dysfunction; CS: cellular senescence; SCE: stem cell exhaustion; AIC: altered intercellular communication; DM: disabled macroautophagy; Inf: inflammation; IA: immunaging. N.A. is not applicable.

**Table 5:** Combined therapies aimed at different aging hallmarks without data on lifespan extension.

**Therapy**	**Reasoning**	**Targeting HA**	**Biological effects**	**Effect of combination**	**Ref.**
**FGF21 + αKlotho + sTGFβR2**	Chosen genes play roles in aging-associated diseases and involved in HA processes, they are involved in separated pathways	AMP, DNS, AIC, IA	Some combinations rescued mice from modeled aging-associated diseases	Synergistic for TGFβ+ FGF21 and TGFβ + αaKlotho; Negative for FGF21 + αaKlotho	[82]
**Ezh2 expression + JmjD3 knockdown** in beta cells of pancreatic islets	EZH2 is decreased in aging b-cells, but it’s OE alone wasn't enough to repress ink4a, which was achieved by adding KD of Trx component JmjD3	EA	Improved beta cell replication	Synergistic in aged mice	[130]
**Oct4, Sox2, Klf4 and c-Myc,** long-term partial induction	Induction of stemness	SCE, EA	Rejuvenation of tissues and whole organism		[121]
**NK-cells infusion + dopamine**	Removing senescent cells	CS	Dopamine enhanced senolytic activity of NK cells	Synergistic	[112]
**Dasatinib + quercetin**	Removing senescent cells	CS	Improve health in aged mice, extends healthspan in progeroid mice	Additive, targets more types of senescent cells than each drug alone	[12]
**Resveratrol + copper**	Elimination of the extracellular chromatin particles	TA, MitD, CS, GI, Inf	Reduced aging-associated pathologies in the cells of aging brain	Synergistic	[126]
**Rapamycin + acarbose + phenylbutyrate**	All components show anti-aging effects and target different but overlapping pathways. Rapamycin inhibits mTOR, acarbose reduce glucose levels, phenylbutyrate improved mice cognitive and physical condition	DNS, Inf, CS, AMP	Delays aging phenotypes, improves healthspan, cognitive and physical condition, reduces tumor burden	Synergistic	[86]
**Dasatinib + Quercetin:** a single 3-day oral treatment regimen with D 100 mg daily and Q 1000 mg total daily (500 mg twice daily)	Removing senescent cells	CS	Decreased senescent cell burden in adipose tissue and epidermis in human with diabetic kidney inflammation	N.A.	[108]
**Growth hormone + metformin + DHEA** (experiment performed on human patients)	rhGH was used based on prior evidence that growth hormone (GH) has thymotrophic and immune reconstituting effects in animals. Because GH might induce hyperinsulinemia which is an undesirable side effect, dehydroepiandrosterone (DHEA) and metformin were added in an attempt to limit the “diabetogenic” effect of GH	EA, DM, LP, DNS	Regenerative response in thymus and bone marrow, delayed epigenetic clock	Synergistic	[131]
**Intra-ocular AAV-delivered Yamanaka factors** (OSK)	Known effect of epigenetic clock reversal	EA	Regeneration of damaged optic nerve, recovery of vision in mice with glaucoma, epigenetic clock reversal	Synergistic	[132]
**Metformin and *Glycyrrhiza uralensis* Fischer extract**	Known anti-obesity agents	DM, LP, DNS	Prevented hepatic steatosis and adiposity, improved glucose homeostasis and reduced inflammation in mice on high-fat diet	Synergistic	[140]
**Dasatinib + quercetin**	Removing senescent cells	CS	Attenuate adipose tissue inflammation, and ameliorate metabolic function (mice)	Synergistic	[141]
**Dasatinib + quercetin**	Removing senescent cells	CS	Ameliorates age-dependent intervertebral disc degeneration in mice	Synergistic	[104]
**Dasatinib + quercetin**	Removing senescent cells	CS	Prevents uterine age-related dysfunction and fibrosis in mice	Synergistic	[106]
**Dasatinib + quercetin:** 100 mg/day + 1250 mg/day; three-days/week over three-weeks orally	Removing senescent cells	CS	Improved physical performance in patients with idiopathic pulmonary fibrosis (IPF)	Synergistic	[109]
**GlyNAC:** glycine (1.33 mmol/kg/day) and N-acetylcysteine (0.81 mmol/kg/day), every 4-weeks for 24-weeks	Metabolism	DNS	GlyNAC supplementation in older adults improves glutathione deficiency, oxidative stress, mitochondrial dysfunction, inflammation, insulin resistance, endothelial dysfunction, genotoxicity, muscle strength, and cognition: Results of a pilot clinical trial	Synergistic	[142]

Abbreviations: GI: genomic instability; TA: telomere attrition; EA: epigenetic alterations; LP: loss of proteostasis; DNS: deregulated nutrient-sensing; MitD: mitochondrial dysfunction; CS: cellular senescence; SCE: stem cell exhaustion; AIC: altered intercellular communication; DM: disabled macroautophagy; Inf: inflammation; IA: immunaging. N.A. is not applicable.

### Combinations that target different hallmarks of aging

The majority of combination therapy studies in mammals focused on complementing therapies that targeted the same hallmark or set of hallmarks of aging (see Table 2). There are only a few exceptions. First, the combination of TP53, p16 and TERT enhancing mutations demonstrated a strong synergistic effect on the lifespan extension [13]. Initial targeting of only two HAs (GI and TA) made mice more resistant to cancer and telomere loss, improved their physical condition, and slowed down the development of aging-associated pathologies and frailty.

Second, a combination gene therapy consisting of three longevity-associated AAV-delivered genes, namely fibroblast growth factor 21 (FGF21), αKlotho and a soluble form of mouse transforming growth factor-β receptor 2 (sTGFβR2) was shown to be beneficial on several mouse models of age-related diseases, such as obesity, type II diabetes, heart failure, and renal failure. Interestingly, the therapy action was synergistic for TGFβ+FGF21 and TGFβ+αKlotho, while the effect was negative for FGF21+αKlotho (the combination worked worse than the individual intervention results for all 4 diseases) [82]. The components of the therapy target different signaling pathways and can be related to the AMP and AIC hallmarks. The triple combination did not outperform either of the two best double combinations by its effects on age-related pathologies. Unfortunately, the effects of these therapies on lifespan were not reported [82].

### Combinations based on rapamycin

Among the pharmacological combination therapies that targeted the same HAs many involved the mTOR inhibitor rapamycin in conjunction with other molecules that affect the DNS hallmark, such as, the antidiabetic inhibitor of liver glucose production metformin, and inhibitor of α-glucosidase acarbose. While metformin and acarbose are readily used as anti-diabetic drugs, chronic rapamycin treatment has been shown to increase glucose intolerance in mice [83, 84]. This is one potential explanation for the increased benefit of the combination. Although some researchers argue that the diabetes-like condition caused by high doses of rapamycin might actually be beneficial in terms of aging by itself.

Combinations of rapamycin and metformin have resulted in an additive effect on mouse lifespan [47], while combinations of rapamycin and acarbose have led to an additive increase in male lifespan with no additive effect in females [85] (if compared to studies where rapamycin [44] or acarbose [75] were used as a monotherapy). One additional study tested a combination of rapamycin, acarbose and phenylbutyrate [86] (a drug that was previously shown to rescue cognitive impairment in a mouse model for Alzheimer diseases) [87]. The combination worked better than individual treatments in decreasing tumor burden as well as cognitive and physical decline in aged mice. Performance tests favored the combination and rapamycin alone comparing to the other two drugs. All drugs reduced the severity of lesions, but the best results were observed for the combination therapy.

Rapamycin and metformin have some overlapping targets and mechanisms of action: mTOR pathway inhibition, enhancing insulin/IGF1 signaling, and autophagy activation. Acarbose affects glucose metabolism by slowing down breakage of polysaccharides to glucose in the intestine. Phenylbutyrate promotes the catabolism of branched-chain amino acids and correspondingly downregulates mTOR [88].

### Combinations of drugs used against cardiovascular disease

A combination of the statin simvastatin and the angiotensin-converting enzyme inhibitor ramipril cause a small yet synergistic increase of male mouse lifespan [89]. Simvastatin lowers low-density lipoprotein (LDL) via reversible inhibition of HMG CoA reductase. Neither of the drugs was shown to increase normal rodent lifespan [43, 101], but they did so in combination. While the mechanisms of action of these drugs are different, they both target similar HAs, namely altered intercellular communication (AIC). One important side-effect of the statin and combined treatment was the increased occurrence of benign lung tumors and hemorrhagic diathesis, but this did not affect overall survival according to the study [89].

It should be noted, that translating any of these findings to humans is particularly difficult because unlike humans mice rarely develop coronary artery atherosclerosis with age, do not exhibit the same high density lipoprotein (HDL) profiles, and have other important difference that affect their cardiovascular system [102].

### Combinations that target senescent cells

The use of senolytics dasatinib + quercetin (D+Q) is another example of the approach where both interventions target the same HA – cellular senescence. Quercetin alone does not improve murine lifespan [103]. The life-extending properties of dasatinib alone have not been investigated, but it was shown that its combination with quercetin is more efficient in elimination of senescent cells and prolongs the lifespan of mice. The oral administration of dasatinib and quercetin decreased the number of naturally occurring senescent cells and their secretion of frailty-associated proinflammatory cytokines in explants of human adipose tissues. Moreover, in both senescent cell–transplanted young mice and naturally aged mice the combination therapy alleviated physical dysfunction and increased post-treatment survival by 36% [104]. Other trials have shown that dasatinib plus quercetin can improve metabolic function, attenuate adipose tissue inflammation and age-dependent intervertebral disc degeneration [105] as well as prevent age-related uterine dysfunction and fibrosis [106].

Dasatinib is a senolytic agent, inhibiting tyrosine kinases, while the antioxidative flavonoid quercetin apart from senolytic activity via inhibition the anti-apoptotic protein Bcl-xL is anti-inflammatory [107]. Additionally, the combination of senolytics dasatinib and quercetin was shown to selectively remove senescent cells in a phase 1 study on nine human patients suffering from diabetic kidney disease [108]. Another study on 14 patients with idiopathic pulmonary fibrosis (IPF) showed that intermittent administration of D+Q slightly but significantly improved their physical functions such as grip strength [109]. In patients with diabetic kidney disease D+Q treatment decreased senescent cell burden in adipose tissue and epidermis [108]. In both human trials a so-called “hit-and-run” administration of senolytics was applied, which is either single dose or intermittent administration. This is rationalized by several reasons: first, senescent phenotypes develop slowly, thus after elimination of senescent cells new ones will not appear soon. Second, the senolytics’ mechanism of action does not require them to be continuously present. Finally, such a regimen was shown to reduce the side effects of dasatinib [110, 111].

Another promising approach to remove senescent cells was demonstrated on a mouse model by applying adoptive infusion of senolytic natural killer (NK) cells boosted by acein, a dopamine-releasing peptide, which caused no senolytic effect by itself. The therapy resulted in reduced local and systemic senescence-associated secretory phenotype (SASP) in aged mice and enhanced the elimination of senescent cells compared to NK cell infusion alone [112].

### Combinations based on calorie restriction

Calorie restriction (CR) is one of the most studied methods of life extension in mice, rats and other mammals [113]. The mechanism of lifespan extension through CR is usually attributed to a number of pathways that involve IGF1/FOXO [114], *mTOR* [115], SIRT1 [116] and other actors. Mice with genetically determined dwarfism (GH, GHR, GHRHR, PROP1 mutations that suppress the GH/IGF1 axis) are well known for their increased lifespan, and there is a number of studies in which such mice were fed with calorie-restricted diets. In most cases such combinations extended the animal’s lifespan even further. For example, PROP1 deficiency alone extended lifespan by 37%, and in combination with CR by up to 69% compared with wild-type ad libitum fed controls [96]. The potential mechanism of lifespan extension in mice by PROP1 deficiency and by CR could be mediated by increased insulin sensitivity, which is further improved by this combination [98].

In rats suppression of GH also extended lifespan, and calorie restriction enhanced this effect [100]. This effect appears to be additive rather than synergistic. The main exceptions are GHR knockout mice that are less responsive to diet restrictions. CR failed to increase overall, median, or average lifespan in these mice and increased maximum lifespan only in females and to a rather small extent (9%) [117]. Also, the combination of GHR KO and CR does not improve insulin sensitivity more than the separate interventions [98].

### Combinations based on partial epigenetic reprogramming

The genes Oct4, Sox2, Klf4 and c-Myc are the four Yamanaka factors (OSKM) that can be used in combination to reprogram specialized cells such as fibroblasts into induced pluripotent cells [118, 119]. Short-term activation of these genes can be used for partial epigenetic reprogramming that does not lead to complete loss of cellular identity but reduces the biological age of targeted cells as measured by epigenetic clocks and other markers of aging [120]. We can classify each OSKM component as aiming for the EA and SCE hallmarks of aging, thus the targets of this therapy overlap.

Virus delivery of the OSK subset of Yamanaka factors to extremely old mice (124 weeks old) decreased their biological age measured by methylation epigenetic clocks, improved their health (decreased frailty) and extended median lifespan by 6% [95]. Another study, which utilized a full set of Yamanaka factors, showed that their long-term partial induction led to the reversion of the epigenetic clock and reduced the expression of numerous genes associated with inflammation, senescence, and stress response, resulting in improved tissue rejuvenation. The phenotype changes were detectable in different tissues, such as the kidney and skin. Epigenetic clock reversal as well as metabolic and transcriptomic changes, including downregulation of pro-inflammatory genes and cellular senescence could be observed after treatment, with the degree of manifestation dependent on the duration of OSKM expression [121].

### Combinations of tumor suppressors

Synergistic lifespan extension can be achieved not just by targeting the same HAs but the same biological processes, e.g., elevated expression of oncosupressors p53 and Arf extended mice median lifespan 16%, while p53 alone only decreased tumor incidence, and Arf had a very weak effect on lifespan [93]. The addition of a TERT transgene extended the lifespan of p53/Arf over-expressing mice by 40%. While p53 and Arf alone protects mice from cancer, neuromuscular degeneration and oxidative damage, addition of TERT attenuated the accumulation of histone markers of double-strand breaks, particularly at telomeric regions, and reduced inflammation [13].

### Combinations that target oxidative stress

According to the free-radical theory of aging, lifespan can be increased by exogenous administration of antioxidants or molecules that enhance the organism’s ability to remove notable oxidants such as H_2_O_2_ [122]. This theory has a number of problems, including observations that certain antioxidants including vitamins A and E may actually increase mortality [123]. One possible explanation is that the introduction of additional antioxidants may negatively affect the endogenous antioxidant systems. Nevertheless, a number of studies tested combinations of interventions aimed at reducing oxidative stress.

One such intervention targeted a single HA (MitD) via overexpression of peroxisomal catalase and superoxide dismutase (PCAT and SOD1 genes) had the synergistic effect on the median but not maximal lifespan [94]. Another study examined the effects of Cu and Zn superoxide dismutase overexpression separately or together, but none of the interventions extended the lifespan of mice [124], contrasting the results of similar experiments on *Drosophila* [125].

Antioxidants are often used to combat aging-related diseases, but there are examples where pro-oxidants provide similar effects. A pro-oxidant combination of chelated copper and resveratrol creates free oxygen radicals which eliminate cell-free chromatin particles originating from dead cell debris that can cause inflammation. Thus, in this case pro-oxidants are anti-inflammatory. Application of the treatment to aged mice led to a reduction of several hallmarks of aging in brain cells including TA, GI, Inf, CS and MitD. The therapy also resulted in significant downregulation of glucose blood levels, cholesterol, amyloid deposition, and C-reactive protein [126].

Another study reported that a combination therapy of glycine and N-acetylcysteine (Gly-NAC) given together significantly prolonged the median and maximum lifespan of mice by 23.7% and 33.1% respectively [91]. Both compounds are claimed to act through ameliorating the age-associated glutathione deficiency and reducing oxidative stress. Glycine itself was previously shown to have only a very modest (4–6%) effect on lifespan [81] while N-acetylcysteine had no effect on female lifespan, but provided a 44% increase in median lifespan in males [127]. We noticed that in the N-acetylcysteine study male mortality appears to be unusually high in the control group, so we doubt that single N-acetylcysteine provided lifespan increase.

Various natural products with presumed antioxidant and anti-inflammatory properties are considered as potential geroprotectors, however many of them do not show any pronounced effects on murine lifespan. The combination of pycnogenol, quercetin and taxifolin, did not expand lifespan of the experimental animals. The same conclusion was made for single treatments of blueberry, pomegranate, green and black tea, cinnamon, sesame, French maritime pine bark, green tea extract, black tea extract and morin. The authors suggest a number of previous reports that these natural products expanding longevity may have resulted from induced caloric restriction [128].

### Combinations that were studied for anti-aging effects other than lifespan extension

Some studies of combination therapies were designed to test if they can reverse separate aging-related phenotypes and/or pathologies without attempting to register any effect on general lifespan.

One combination gene therapy against type II diabetes was aimed at overexpressing the transgenic EZH2 protein, a key component of the Polycomb complex, while simultaneously knocking down the *MII1* gene, a part of the Trithorax complex. It resulted in successful repression of the Ink4a locus and rejuvenation of the pancreatic islets through promotion of β-cell proliferation. Notably, the EZH2 alone was not sufficient to cause improvements in mice older than 8 months while simultaneous *MII1* knockdown acted well even in aged animals. Additionally, a combination of EZH2 overexpression and JnjD3 knockdown in beta cells of aged mice enhanced replication of beta cells, unlike the separate interventions [130].

There is also limited evidence that some combination therapies can ameliorate age-related phenotypes in humans. For example, a combination of growth hormone (GH), dehydroepiandrosterone (DHEA) and metformin was used on a group of 10 patients aged 51–65 years. GH was aimed at thymus regeneration and reduction of immunosenescence, while two other drugs were supposed to reduce the diabetogenic side effects. Indeed, the therapy resulted in regenerative response in thymus and bone marrow and delayed epigenetic clock advancement in the studied tissues [131].

A prominent study used a combination of three Yamanaka factors (excluding the carcinogenic c-Myc) to regenerate damaged optic nerves and recover vision in mice with glaucoma served epigenetic age reversal. No pronounced side effects were observed [132].

### Effect of combination therapies on lifespan of progeroid and disease animal models

Although our review focuses mainly on experiments where therapeutic interventions were able to significantly expand the lifespan of normally aging lab mice, we decided to make a separate overview of combination therapies which prolonged lifespan and healthspan in progeroid and other rapidly aging mouse models. Despite progeroid models being less representative of the normal aging processes, studies on them still remain an important source of knowledge about the mechanisms of aging and anti-aging treatments [133].

In a well-known Hutchinson-Gilford Zmpste24 −/− progeria mouse model of premature aging, a combination of three synergistically acting drugs targeting the AMP hallmark (by preventing vascular calcification) has shown a significant though moderate median lifespan increase. The application of extracellular ATP yielding pyrophosphate (which is a known inhibitor of calcium-phosphate deposition and therefore of vascular calcification [134]) was boosted by two drugs: tissue-nonspecific alkaline phosphatase (TNAP) inhibitor levamisole and ectonucleoside triphosphate diphosphohydrolase (eNTPD) inhibitor ARL67156. The drugs enhanced the production of pyrophosphate from ATP and reduced its hydrolysis, respectively, leading to prevention of vascular calcification. Notably, ATP alone did not play any effect on the lifespan of studied animals [135].

A combined treatment with statins (pravastatin) and aminobisphosphonates (zolendronate) was enough to synergistically extend longevity in the same mouse model. Both drugs target the same hallmark of aging, namely the loss of proteostasis, by simultaneously inhibiting two interchangeable pathways of prelamin A and progerin prenylation. The therapy significantly prolonged median and maximum lifespan (Table 4) and evidently improved the aging-like phenotypes typical for the model, including growth retardation, loss of weight, lipodystrophy, hair loss and accumulation of bone defects [136].

Another progeroid mouse model was efficiently treated by the combination of Yamanaka factors (OSKM). LAKI mice carrying a truncating lamin mutation were subjected to short-term cyclic OSKM induction, which resulted in amelioration of cellular and physiological hallmarks of aging and led to an extension in median and maximal (up to 20%) lifespan [137].

Autoimmunity is a well-known age-dependent pathology. Studies of autoimmune responses often involve the autoimmunity prone NZB × NZW F1 mice model. On this model the additive effect of calorie restriction and fish oil, targeting the DNS and INF hallmarks was observed. The combination was shown to reduce levels of several important proinflammatory factors, including TNF-α and NF-kB and to upregulate antioxidant pathways leading to a dramatic median lifespan increase of 166% [138]. Another experiment on the same model involved mice treatment with a known immunosuppressant cyclophosphamide along with an immunostimulator tilorone. This combination of drugs exhibited an additive effect on lifespan extension [139].

### Developing combination therapies to enhance mammalian longevity

It appears that combination therapy is a promising approach for mammalian lifespan extension. It can work when targeted hallmarks of aging are different and even in cases when the presumed hallmarks overlap. However, the number of tested combinations of longevity-enhancing treatments is relatively small and most studies are done in rodents. The design and implementation of additional studies is warranted, including those in other species of mammals.

**Figure 1:**
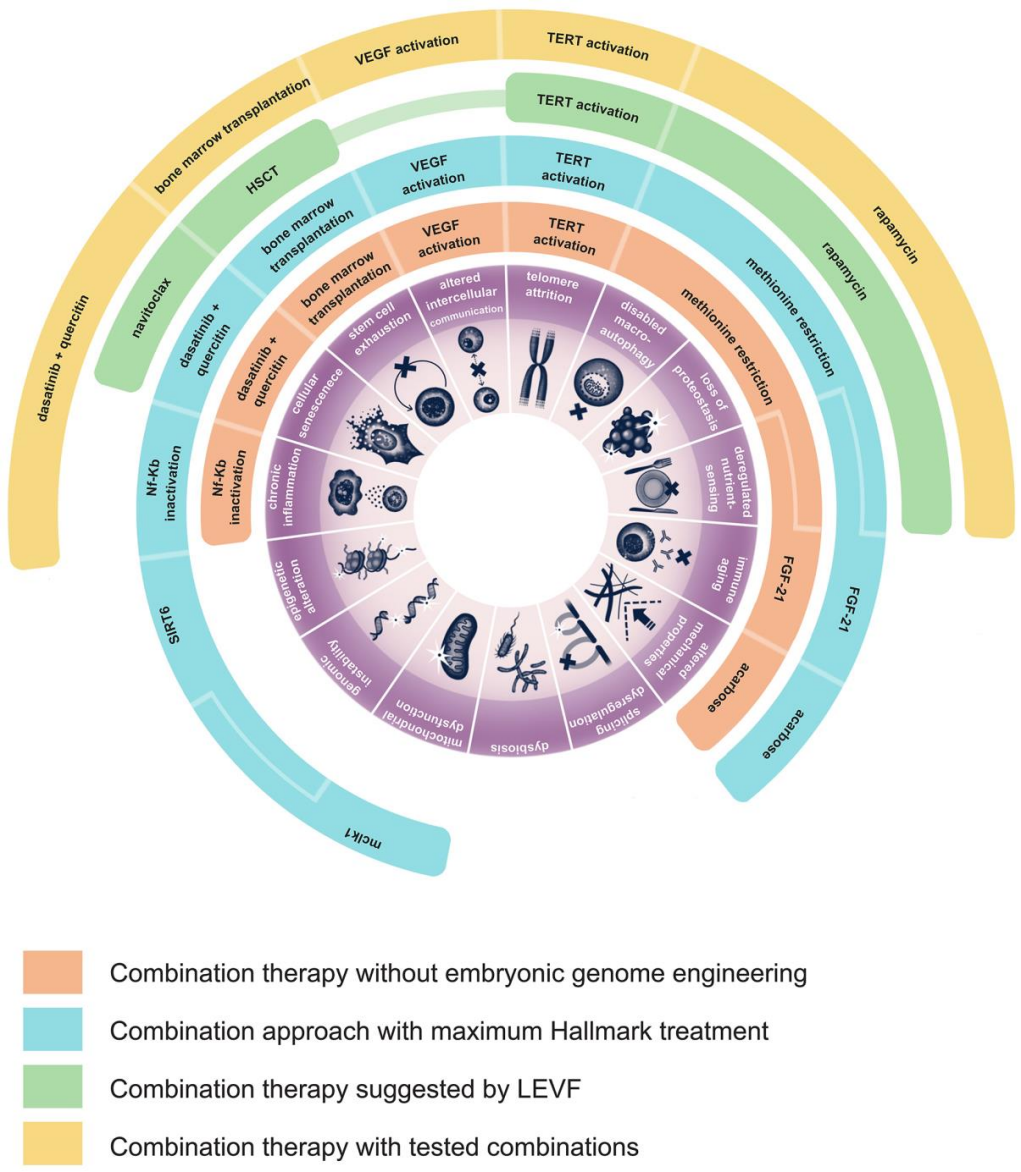
**Possible strategies of development of aging combination therapies.** This figure illustrates the various hallmarks of aging that could be addressed by different existing interventions. Additionally, it proposes four example combinations of therapies, each detailing which specific treatment targets a particular hallmark of aging.

The current availability and diversity of lifespan promoting therapies allows the possibility to design studies in mice targeting at least thirteen hallmarks of aging simultaneously. Nine of these hallmarks can be readily targeted in animals of any genetic background. For example, this can be accomplished with a combination of dasatinib plus quercetin, FGF-21, acarbose, methionine restriction, young bone marrow transplantation and gene therapy for VEGF and TERT activation and hypothalamus-restricted Nf-Kb inactivation (example combination 1, Figure 1). Three more hallmarks can be targeted by genetically engineering mice overexpressing SIRT6 and by creating mclk1 knockout heterozygotes (example combination 2, Figure 1). Theoretically the same effects can be produced by experimental gene therapies. However, example combinations 1 and 2 involve surgical procedures that by themselves can be traumatizing, a lentiviral gene therapy that can cause insertional mutagenesis, a CMV gene therapy that in theory may contribute to immune system depletion, an AAV gene therapy that like the other two gene therapies may cause immune reactions and liver toxicity.

Recently the LEVF foundation initiated an experiment to test a combined therapy of rapamycin, a senolytic called navitoclax, hematopoietic stem cell transplantation (HSCT), and telomerase expression (example combination 3, Figure 1) [143]. This approach would target six hallmarks of aging: deregulated nutrient sensing (DNS), disabled macroautophagy (DM), loss of proteostasis (LP), cellular senescence (CS), stem cell exhaustion (SCE) and telomere attrition (TA).

Given that there are many possible interventions that have provided lifespan extension in mice as single therapies, for the conservation of effort and resources, it would be reasonable to develop strategies for prioritizing the most promising combined therapies. Ideally, these strategies should take into account the cost and effectiveness of individual treatments, their theoretical interactions and whether the targeted hallmarks overlap. We would also argue that we should prioritize interventions that can be applied to already aged animals given the ultimate practical goal of prolonging the lifespan of existing humans. Perhaps interventions that are evidently less effective in humans than in mice, dangerous or difficult to sustain should be deprioritized for practical purposes. For example, calorie restriction without malnutrition while being highly effective at extending mouse lifespan is unlikely to provide proportional life extension in humans and might be difficult to follow [144]. Indeed, it is currently unclear whether calorie restriction in the true sense (i.e., not rescue from overeating/obesity) has any of the effects seen in rodents when it comes to humans.

Taking these considerations into account, one strategy could be to combine therapies that provided the highest lifespan extension as individual treatments. In descending order these would be (VEGF gene therapy up to +49% median), methionine restriction (up to +42% mean), TERT gene therapy (up to +41% median), follistatin gene therapy (+32% mean) and bone marrow transplantation (up to +28% mean). Note that the provided value of increased lifespan expectancy for bone marrow transplantation might be inflated given the high mortality in one of the studies’ control groups. A more conservative result of +12% median lifespan increase was reported in a more recent study [64].

Another strategy is to select the smallest number of treatments that cover the largest number of aging hallmarks (while still preferring treatments with highest lifespan extension values). For example, dasatinib + quercetin (CS + inf), rapamycin (DM + LP + DNS), VEGF gene therapy (AIC), bone marrow transplantation (SCE) and telomerase gene therapy (TA), perhaps with the addition of acarbose. Unfortunately, as mentioned earlier, we were unable to identify interventions that have confirmed positive effects on lifespan and can be applied to adult animals for several hallmarks of aging.

A third strategy is to start with the best existing combinations and expand them with interventions that provide the highest lifespan extension as an individual treatment but were not used before, preferably targeting a hallmark of aging that the initial combination did not. In this case we would recommend starting with rapamycin + acarbose (up to +37% lifespan extension) followed by the individual combinations from the first strategy.

A fourth strategy may be based on systematic screening for the synergistically best pairs of combinations that target different hallmarks of aging. This approach might also elucidate conflicting impacts of interventions that can offset or even worsen each other’s effects. The absence of an additive and synergistic effect may indicate, in particular, that the downstream effects of two therapies overlap.

Finally, a fifth strategy might consider investigating combinations that consist of widely-used molecular components with well-documented safety profiles in humans. Such combinations might include acarbose, glycine, taurine, and spermidine.

It’s worth noting that some combinations might be considered more practically feasible in real-life clinical settings. For example, small molecule drugs and supplements are easier to use than gene therapies, followed by bone-marrow transplantation. However, the widespread use of gene therapies by the general public is possible, given the availability of COVID vaccines which are similar in design. Multiple gene therapies have successfully finished human clinical trials and are available for a wide range of diseases from hemophilia A and B, to RPE65-mutation-associated retinal dystrophy, dystrophic epidermolysis bullosa and certain types of cancer [145]. Given the large amount of genes potentially involved in aging such as those reviewed in comprehensive Open genes database [146], we believe gene therapies are desirable and viable components of future anti-aging combination therapies.

### Further directions

We observe that a number of genetic mutations increase the lifespan of mammals, but the effects of these mutations were not tested as possible longevity enhancing gene therapies. This includes gene therapies to overexpress Sirt6, Sirt1, p53 (+ p16 + Arf), FoxM1, catalase OE in peroxisomes + superoxide dismutase and the reduction of expression of GH, GHR, GHRHR, Prop1, mclk, macrophage inhibitory factor, IGF1R and Becn1F121A/F121A mutation in beclin 1. Individual testing of these approaches could provide novel single interventions that could be combined with future effective combination therapies. The combination of FGF21, αKlotho and sTGFβR2 deserves deeper investigation in terms of lifespan prolongation as a promising combination gene therapy that uses a number of genes that were all previously described as healthspan- or lifespan-promoting [51, 71, 147, 148].

There are some genetic and pharmacological interventions that are promising but, to our surprise, have not been tested in mice. One of them is the upregulation of FOXO3a, which is linked to human longevity and healthspan [149–152]. Some existing FOXO3a gene variants are associated with extreme longevity in human populations [149]. It is also known to be a positive regulator of healthspan, that affects the aging of the cardiovascular system [153]. The lifespan-expanding function of FoxO proteins is highly evolutionary conserved: the single FoxO homolog in *Hydra* contributes to the anti-aging mechanisms of this freshwater polyp [154, 155]. The prolongevity effects of homologous genes have been vastly studied in *C. elegans*, where they are associated with the DNS HA [156]. FOXO3 mediates the life-extending effect of CR in mice [114], but we haven’t found any studies where it was specifically overexpressed in mammals and lifespan changes were assessed.

### Limitations

One important limitation of our analysis is that the effects of treatments on lifespan or other aging-related metrics in mice are at least to some extent dependent upon the conditions in which the animals are maintained and the genetic background of the strains used. Long-term breeding in laboratories has led to some strains being mildly progeroid, thus some of the life-extending effects could result from the rescue of the underlying conditions [157, 158]. This may further reduce the translatability of pro-longevity treatments in mice to other animals including humans. Additional studies in other species, such as dogs, could help elucidate the pro-longevity treatments that are more generalizable.

Another limitation comes from the hallmarks of aging. While currently we do not have a better framework for describing the aging process, the hallmarks of aging have relatively limited explanatory value regarding the general etiologies of aging and do not discern the primary and secondary causes of aging [20]. As we understand more about the aging process, we will be able to provide a better framework for selecting the most efficacious combination treatments.

There is also the question whether aging can truly be intervened. We believe it is possible given that the lifespans of many model organisms ranging from roundworms to mice have been greatly improved. The potential for human life extension is also supported by the increase of human lifespan during the course of evolution, when compared to our ancient ancestors. Finally, the existence of negligible senescence in some mammals, such as the naked mole rat suggests that aging is not an insurmountable challenge for life [2].

## CONCLUSIONS

Aging is a complex problem that most likely requires multiple simultaneous solutions. Several combined therapies that target multiple different hallmarks of aging or even the same hallmarks of aging have been shown to provide additive and sometimes synergistic effects on mammalian lifespan. Nevertheless, most tested combined therapies involved targeting of only a small fraction of aging hallmarks and many potentially beneficial combinations have not been tested. We propose several strategies to select potential combination therapies against aging. These strategies include the idea of targeting as many hallmarks of aging as possible, prioritizing single interventions that provide the highest increase in lifespan, or adding single interventions to existing combinations with highest contributions to lifespan. We believe that the use of combination therapy is not only feasible in the field of longevity but provides the best practical opportunity to prevent age-related pathologies and enhance human lifespan in the nearest future.

## References

[1] Ledberg A. Exponential increase in mortality with age is a generic property of a simple model system of damage accumulation and death. PLoS One. 2020; 15:e0233384. 10.1371/journal.pone.0233384 32497107 PMC7272078

[2] Buffenstein R. Negligible senescence in the longest living rodent, the naked mole-rat: insights from a successfully aging species. J Comp Physiol B. 2008; 178:439–45. 10.1007/s00360-007-0237-5 18180931

[3] Antiretroviral Therapy Cohort Collaboration. Survival of HIV-positive patients starting antiretroviral therapy between 1996 and 2013: a collaborative analysis of cohort studies. Lancet HIV. 2017; 4:e349–56. 10.1016/S2352-3018(17)30066-8 28501495 PMC5555438

[4] Bayat Mokhtari R, Homayouni TS, Baluch N, Morgatskaya E, Kumar S, Das B, Yeger H. Combination therapy in combating cancer. Oncotarget. 2017; 8:38022–43. 10.18632/oncotarget.16723 28410237 PMC5514969

[5] Chen SH, Lahav G. Two is better than one; toward a rational design of combinatorial therapy. Curr Opin Struct Biol. 2016; 41:145–50. 10.1016/j.sbi.2016.07.020 27521655 PMC5469547

[6] Tängdén T. Combination antibiotic therapy for multidrug-resistant Gram-negative bacteria. Ups J Med Sci. 2014; 119:149–53. 10.3109/03009734.2014.899279 24666223 PMC4034552

[7] Paz-Ares L, Luft A, Vicente D, Tafreshi A, Gümüş M, Mazières J, Hermes B, Çay Şenler F, Csőszi T, Fülöp A, Rodríguez-Cid J, Wilson J, Sugawara S, et al, and KEYNOTE-407 Investigators. Pembrolizumab plus Chemotherapy for Squamous Non-Small-Cell Lung Cancer. N Engl J Med. 2018; 379:2040–51. 10.1056/NEJMoa1810865 30280635

[8] Gravara LD, Battiloro C, Cantile R, Letizia A, Vitiello F, Montesarchio V, Rocco D. Chemotherapy and/or immune checkpoint inhibitors in NSCLC first-line setting: what is the best approach? Lung Cancer Manag. 2020; 9:LMT22. 10.2217/lmt-2019-0018 32256708 PMC7110571

[9] Chen D, Li PW, Goldstein BA, Cai W, Thomas EL, Chen F, Hubbard AE, Melov S, Kapahi P. Germline signaling mediates the synergistically prolonged longevity produced by double mutations in daf-2 and rsks-1 in C. elegans. Cell Rep. 2013; 5:1600–10. 10.1016/j.celrep.2013.11.018 24332851 PMC3904953

[10] Castillo-Quan JI, Tain LS, Kinghorn KJ, Li L, Grönke S, Hinze Y, Blackwell TK, Bjedov I, Partridge L. A triple drug combination targeting components of the nutrient-sensing network maximizes longevity. Proc Natl Acad Sci U S A. 2019; 116:20817–9. 10.1073/pnas.1913212116 31570569 PMC6800352

[11] Kaur P, Otgonbaatar A, Ramamoorthy A, Chua EHZ, Harmston N, Gruber J, Tolwinski NS. Combining stem cell rejuvenation and senescence targeting to synergistically extend lifespan. Aging (Albany NY). 2022; 14:8270–91. 10.18632/aging.204347 36287172 PMC9648810

[12] Zhu Y, Tchkonia T, Pirtskhalava T, Gower AC, Ding H, Giorgadze N, Palmer AK, Ikeno Y, Hubbard GB, Lenburg M, O'Hara SP, LaRusso NF, Miller JD, et al. The Achilles' heel of senescent cells: from transcriptome to senolytic drugs. Aging Cell. 2015; 14:644–58. 10.1111/acel.12344 25754370 PMC4531078

[13] Tomás-Loba A, Flores I, Fernández-Marcos PJ, Cayuela ML, Maraver A, Tejera A, Borrás C, Matheu A, Klatt P, Flores JM, Viña J, Serrano M, Blasco MA. Telomerase reverse transcriptase delays aging in cancer-resistant mice. Cell. 2008; 135:609–22. 10.1016/j.cell.2008.09.034 19013273

[14] Bernardes de Jesus B, Vera E, Schneeberger K, Tejera AM, Ayuso E, Bosch F, Blasco MA. Telomerase gene therapy in adult and old mice delays aging and increases longevity without increasing cancer. EMBO Mol Med. 2012; 4:691–704. 10.1002/emmm.201200245 22585399 PMC3494070

[15] Jaijyan DK, Selariu A, Cruz-Cosme R, Tong M, Yang S, Stefa A, Kekich D, Sadoshima J, Herbig U, Tang Q, Church G, Parrish EL, Zhu H. New intranasal and injectable gene therapy for healthy life extension. Proc Natl Acad Sci U S A. 2022; 119:e2121499119. 10.1073/pnas.2121499119 35537048 PMC9171804

[16] Bunu G, Toren D, Ion CF, Barardo D, Sârghie L, Grigore LG, de Magalhães JP, Fraifeld VE, Tacutu R. SynergyAge, a curated database for synergistic and antagonistic interactions of longevity-associated genes. Sci Data. 2020; 7:366. 10.1038/s41597-020-00710-z 33106474 PMC7589469

[17] López-Otín C, Blasco MA, Partridge L, Serrano M, Kroemer G. The hallmarks of aging. Cell. 2013; 153:1194–217. 10.1016/j.cell.2013.05.039 23746838 PMC3836174

[18] Schmauck-Medina T, Molière A, Lautrup S, Zhang J, Chlopicki S, Madsen HB, Cao S, Soendenbroe C, Mansell E, Vestergaard MB, Li Z, Shiloh Y, Opresko PL, et al. New hallmarks of ageing: a 2022 Copenhagen ageing meeting summary. Aging (Albany NY). 2022; 14:6829–39. 10.18632/aging.204248 36040386 PMC9467401

[19] Urfer SR, Kaeberlein TL, Mailheau S, Bergman PJ, Creevy KE, Promislow DEL, Kaeberlein M. A randomized controlled trial to establish effects of short-term rapamycin treatment in 24 middle-aged companion dogs. Geroscience. 2017; 39:117–27. 10.1007/s11357-017-9972-z 28374166 PMC5411365

[20] Gems D, de Magalhães JP. The hoverfly and the wasp: A critique of the hallmarks of aging as a paradigm. Ageing Res Rev. 2021; 70:101407. 10.1016/j.arr.2021.101407 34271186 PMC7611451

[21] López-Otín C, Blasco MA, Partridge L, Serrano M, Kroemer G. Hallmarks of aging: An expanding universe. Cell. 2023; 186:243–78. 10.1016/j.cell.2022.11.001 36599349

[22] Fedintsev A, Moskalev A. Stochastic non-enzymatic modification of long-lived macromolecules - A missing hallmark of aging. Ageing Res Rev. 2020; 62:101097. 10.1016/j.arr.2020.101097 32540391

[23] Palmer DB. The effect of age on thymic function. Front Immunol. 2013; 4:316. 10.3389/fimmu.2013.00316 24109481 PMC3791471

[24] Hirst JA, Farmer AJ, Ali R, Roberts NW, Stevens RJ. Quantifying the effect of metformin treatment and dose on glycemic control. Diabetes Care. 2012; 35:446–54. 10.2337/dc11-1465 22275444 PMC3263873

[25] Tazume S, Umehara K, Matsuzawa H, Aikawa H, Hashimoto K, Sasaki S. Effects of germfree status and food restriction on longevity and growth of mice. Jikken Dobutsu. 1991; 40:517–22. 10.1538/expanim1978.40.4_517 1748169

[26] Bárcena C, Valdés-Mas R, Mayoral P, Garabaya C, Durand S, Rodríguez F, Fernández-García MT, Salazar N, Nogacka AM, Garatachea N, Bossut N, Aprahamian F, Lucia A, et al. Healthspan and lifespan extension by fecal microbiota transplantation into progeroid mice. Nat Med. 2019; 25:1234–42. 10.1038/s41591-019-0504-5 31332389

[27] Matsumoto M, Kurihara S, Kibe R, Ashida H, Benno Y. Longevity in mice is promoted by probiotic-induced suppression of colonic senescence dependent on upregulation of gut bacterial polyamine production. PLoS One. 2011; 6:e23652. 10.1371/journal.pone.0023652 21858192 PMC3156754

[28] Kaeberlein M. The Biology of Aging: Citizen Scientists and Their Pets as a Bridge Between Research on Model Organisms and Human Subjects. Vet Pathol. 2016; 53:291–8. 10.1177/0300985815591082 26077786 PMC4794982

[29] Wilkinson JE, Burmeister L, Brooks SV, Chan CC, Friedline S, Harrison DE, Hejtmancik JF, Nadon N, Strong R, Wood LK, Woodward MA, Miller RA. Rapamycin slows aging in mice. Aging Cell. 2012; 11:675–82. 10.1111/j.1474-9726.2012.00832.x 22587563 PMC3434687

[30] Infante M, Leoni M, Caprio M, Fabbri A. Long-term metformin therapy and vitamin B12 deficiency: An association to bear in mind. World J Diabetes. 2021; 12:916–31. 10.4239/wjd.v12.i7.916 34326945 PMC8311483

[31] Nekoukar Z, Moghimi M, Salehifar E. A narrative review on adverse effects of dasatinib with a focus on pharmacotherapy of dasatinib-induced pulmonary toxicities. Blood Res. 2021; 56:229–42. 10.5045/br.2021.2021117 34776414 PMC8721448

[32] Kelberman D, Turton JP, Woods KS, Mehta A, Al-Khawari M, Greening J, Swift PG, Otonkoski T, Rhodes SJ, Dattani MT. Molecular analysis of novel PROP1 mutations associated with combined pituitary hormone deficiency (CPHD). Clin Endocrinol (Oxf). 2009; 70:96–103. 10.1111/j.1365-2265.2008.03326.x 19128366

[33] Bertko E, Klammt J, Dusatkova P, Bahceci M, Gonc N, Ten Have L, Kandemir N, Mansmann G, Obermannova B, Oostdijk W, Pfäffle H, Rockstroh-Lippold D, Schlicke M, et al. Combined pituitary hormone deficiency due to gross deletions in the POU1F1 (PIT-1) and PROP1 genes. J Hum Genet. 2017; 62:755–62. 10.1038/jhg.2017.34 28356564 PMC5537413

[34] Aguiar-Oliveira MH, Bartke A. Growth Hormone Deficiency: Health and Longevity. Endocr Rev. 2019; 40:575–601. 10.1210/er.2018-00216 30576428 PMC6416709

[35] Colella P, Ronzitti G, Mingozzi F. Emerging Issues in AAV-Mediated *In Vivo* Gene Therapy. Mol Ther Methods Clin Dev. 2017; 8:87–104. 10.1016/j.omtm.2017.11.007 29326962 PMC5758940

[36] Ertl HCJ. Immunogenicity and toxicity of AAV gene therapy. Front Immunol. 2022; 13:975803. 10.3389/fimmu.2022.975803 36032092 PMC9411526

[37] Bohne J, Cathomen T. Genotoxicity in gene therapy: an account of vector integration and designer nucleases. Curr Opin Mol Ther. 2008; 10:214–23. 18535928

[38] Hacein-Bey-Abina S, Garrigue A, Wang GP, Soulier J, Lim A, Morillon E, Clappier E, Caccavelli L, Delabesse E, Beldjord K, Asnafi V, MacIntyre E, Dal Cortivo L, et al. Insertional oncogenesis in 4 patients after retrovirus-mediated gene therapy of SCID-X1. J Clin Invest. 2008; 118:3132–42. 10.1172/JCI35700 18688285 PMC2496963

[39] Yousefzadeh MJ, Zhu Y, McGowan SJ, Angelini L, Fuhrmann-Stroissnigg H, Xu M, Ling YY, Melos KI, Pirtskhalava T, Inman CL, McGuckian C, Wade EA, Kato JI, et al. Fisetin is a senotherapeutic that extends health and lifespan. EBioMedicine. 2018; 36:18–28. 10.1016/j.ebiom.2018.09.015 30279143 PMC6197652

[40] Baker DJ, Childs BG, Durik M, Wijers ME, Sieben CJ, Zhong J, Saltness RA, Jeganathan KB, Verzosa GC, Pezeshki A, Khazaie K, Miller JD, van Deursen JM. Naturally occurring p16(Ink4a)-positive cells shorten healthy lifespan. Nature. 2016; 530:184–9. 10.1038/nature16932 26840489 PMC4845101

[41] Bitto A, Ito TK, Pineda VV, LeTexier NJ, Huang HZ, Sutlief E, Tung H, Vizzini N, Chen B, Smith K, Meza D, Yajima M, Beyer RP, et al. Transient rapamycin treatment can increase lifespan and healthspan in middle-aged mice. Elife. 2016; 5:e16351. 10.7554/eLife.16351 27549339 PMC4996648

[42] Strong R, Miller RA, Bogue M, Fernandez E, Javors MA, Libert S, Marinez PA, Murphy MP, Musi N, Nelson JF, Petrascheck M, Reifsnyder P, Richardson A, et al. Rapamycin-mediated mouse lifespan extension: Late-life dosage regimes with sex-specific effects. Aging Cell. 2020; 19:e13269. 10.1111/acel.13269 33145977 PMC7681050

[43] Miller RA, Harrison DE, Astle CM, Baur JA, Boyd AR, de Cabo R, Fernandez E, Flurkey K, Javors MA, Nelson JF, Orihuela CJ, Pletcher S, Sharp ZD, et al. Rapamycin, but not resveratrol or simvastatin, extends life span of genetically heterogeneous mice. J Gerontol A Biol Sci Med Sci. 2011; 66:191–201. 10.1093/gerona/glq178 20974732 PMC3021372

[44] Miller RA, Harrison DE, Astle CM, Fernandez E, Flurkey K, Han M, Javors MA, Li X, Nadon NL, Nelson JF, Pletcher S, Salmon AB, Sharp ZD, et al. Rapamycin-mediated lifespan increase in mice is dose and sex dependent and metabolically distinct from dietary restriction. Aging Cell. 2014; 13:468–77. 10.1111/acel.12194 24341993 PMC4032600

[45] Shindyapina AV, Cho Y, Kaya A, Tyshkovskiy A, Castro JP, Deik A, Gordevicius J, Poganik JR, Clish CB, Horvath S, Peshkin L, Gladyshev VN. Rapamycin treatment during development extends life span and health span of male mice and *Daphnia magna*. Sci Adv. 2022; 8:eabo5482. 10.1126/sciadv.abo5482 36112674 PMC9481125

[46] Harrison DE, Strong R, Sharp ZD, Nelson JF, Astle CM, Flurkey K, Nadon NL, Wilkinson JE, Frenkel K, Carter CS, Pahor M, Javors MA, Fernandez E, Miller RA. Rapamycin fed late in life extends lifespan in genetically heterogeneous mice. Nature. 2009; 460:392–5. 10.1038/nature08221 19587680 PMC2786175

[47] Strong R, Miller RA, Antebi A, Astle CM, Bogue M, Denzel MS, Fernandez E, Flurkey K, Hamilton KL, Lamming DW, Javors MA, de Magalhães JP, Martinez PA, et al. Longer lifespan in male mice treated with a weakly estrogenic agonist, an antioxidant, an α-glucosidase inhibitor or a Nrf2-inducer. Aging Cell. 2016; 15:872–84. 10.1111/acel.12496 27312235 PMC5013015

[48] Martin-Montalvo A, Mercken EM, Mitchell SJ, Palacios HH, Mote PL, Scheibye-Knudsen M, Gomes AP, Ward TM, Minor RK, Blouin MJ, Schwab M, Pollak M, Zhang Y, et al. Metformin improves healthspan and lifespan in mice. Nat Commun. 2013; 4:2192. 10.1038/ncomms3192 23900241 PMC3736576

[49] Richie JP Jr, Leutzinger Y, Parthasarathy S, Malloy V, Orentreich N, Zimmerman JA. Methionine restriction increases blood glutathione and longevity in F344 rats. FASEB J. 1994; 8:1302–7. 10.1096/fasebj.8.15.8001743 8001743

[50] Blüher M, Kahn BB, Kahn CR. Extended longevity in mice lacking the insulin receptor in adipose tissue. Science. 2003; 299:572–4. 10.1126/science.1078223 12543978

[51] Kurosu H, Yamamoto M, Clark JD, Pastor JV, Nandi A, Gurnani P, McGuinness OP, Chikuda H, Yamaguchi M, Kawaguchi H, Shimomura I, Takayama Y, Herz J, et al. Suppression of aging in mice by the hormone Klotho. Science. 2005; 309:1829–33. 10.1126/science.1112766 16123266 PMC2536606

[52] Asadi Shahmirzadi A, Edgar D, Liao CY, Hsu YM, Lucanic M, Asadi Shahmirzadi A, Wiley CD, Gan G, Kim DE, Kasler HG, Kuehnemann C, Kaplowitz B, Bhaumik D, et al. Alpha-Ketoglutarate, an Endogenous Metabolite, Extends Lifespan and Compresses Morbidity in Aging Mice. Cell Metab. 2020; 32:447–56.e6. 10.1016/j.cmet.2020.08.004 32877690 PMC8508957

[53] Conti B, Sanchez-Alavez M, Winsky-Sommerer R, Morale MC, Lucero J, Brownell S, Fabre V, Huitron-Resendiz S, Henriksen S, Zorrilla EP, de Lecea L, Bartfai T. Transgenic mice with a reduced core body temperature have an increased life span. Science. 2006; 314:825–8. 10.1126/science.1132191 17082459

[54] Weindruch R, Walford RL, Fligiel S, Guthrie D. The retardation of aging in mice by dietary restriction: longevity, cancer, immunity and lifetime energy intake. J Nutr. 1986; 116:641–54. 10.1093/jn/116.4.641 3958810

[55] Holzenberger M, Dupont J, Ducos B, Leneuve P, Géloën A, Even PC, Cervera P, Le Bouc Y. IGF-1 receptor regulates lifespan and resistance to oxidative stress in mice. Nature. 2003; 421:182–7. 10.1038/nature01298 12483226

[56] Grunewald M, Kumar S, Sharife H, Volinsky E, Gileles-Hillel A, Licht T, Permyakova A, Hinden L, Azar S, Friedmann Y, Kupetz P, Tzuberi R, Anisimov A, et al. Counteracting age-related VEGF signaling insufficiency promotes healthy aging and extends life span. Science. 2021; 373:eabc8479. 10.1126/science.abc8479 34326210

[57] Fernández ÁF, Sebti S, Wei Y, Zou Z, Shi M, McMillan KL, He C, Ting T, Liu Y, Chiang WC, Marciano DK, Schiattarella GG, Bhagat G, et al. Disruption of the beclin 1-BCL2 autophagy regulatory complex promotes longevity in mice. Nature. 2018; 558:136–40. 10.1038/s41586-018-0162-7 29849149 PMC5992097

[58] Benigni A, Corna D, Zoja C, Sonzogni A, Latini R, Salio M, Conti S, Rottoli D, Longaretti L, Cassis P, Morigi M, Coffman TM, Remuzzi G. Disruption of the Ang II type 1 receptor promotes longevity in mice. J Clin Invest. 2009; 119:524–30. 10.1172/JCI36703 19197138 PMC2648681

[59] Landisman CE, Connors BW. Long-term modulation of electrical synapses in the mammalian thalamus. Science. 2005; 310:1809–13. 10.1126/science.1114655 16357260

[60] Flurkey K, Papaconstantinou J, Harrison DE. The Snell dwarf mutation Pit1(dw) can increase life span in mice. Mech Ageing Dev. 2002; 123:121–30. 10.1016/s0047-6374(01)00339-6 11718806

[61] Flurkey K, Papaconstantinou J, Miller RA, Harrison DE. Lifespan extension and delayed immune and collagen aging in mutant mice with defects in growth hormone production. Proc Natl Acad Sci U S A. 2001; 98:6736–41. 10.1073/pnas.111158898 11371619 PMC34422

[62] Migliaccio E, Giorgio M, Mele S, Pelicci G, Reboldi P, Pandolfi PP, Lanfrancone L, Pelicci PG. The p66shc adaptor protein controls oxidative stress response and life span in mammals. Nature. 1999; 402:309–13. 10.1038/46311 10580504

[63] Kovina MV, Karnaukhov AV, Krasheninnikov ME, Kovin AL, Gazheev ST, Sergievich LA, Karnaukhova EV, Bogdanenko EV, Balyasin MV, Khodarovich YM, Dyuzheva TG, Lyundup AV. Extension of Maximal Lifespan and High Bone Marrow Chimerism After Nonmyeloablative Syngeneic Transplantation of Bone Marrow From Young to Old Mice. Front Genet. 2019; 10:310. 10.3389/fgene.2019.00310 31031800 PMC6473025

[64] Guderyon MJ, Chen C, Bhattacharjee A, Ge G, Fernandez RA, Gelfond JAL, Gorena KM, Cheng CJ, Li Y, Nelson JF, Strong RJ, Hornsby PJ, Clark RA, Li S. Mobilization-based transplantation of young-donor hematopoietic stem cells extends lifespan in mice. Aging Cell. 2020; 19:e13110. 10.1111/acel.13110 32012439 PMC7059148

[65] Kanfi Y, Naiman S, Amir G, Peshti V, Zinman G, Nahum L, Bar-Joseph Z, Cohen HY. The sirtuin SIRT6 regulates lifespan in male mice. Nature. 2012; 483:218–21. 10.1038/nature10815 22367546

[66] Roichman A, Elhanati S, Aon MA, Abramovich I, Di Francesco A, Shahar Y, Avivi MY, Shurgi M, Rubinstein A, Wiesner Y, Shuchami A, Petrover Z, Lebenthal-Loinger I, et al. Restoration of energy homeostasis by SIRT6 extends healthy lifespan. Nat Commun. 2021; 12:3208. 10.1038/s41467-021-23545-7 34050173 PMC8163764

[67] Shyu YC, Liao PC, Huang TS, Yang CJ, Lu MJ, Huang SM, Lin XY, Liou CC, Kao YH, Lu CH, Peng HL, Chen JR, Cherng WJ, et al. Genetic Disruption of KLF1 K74 SUMOylation in Hematopoietic System Promotes Healthy Longevity in Mice. Adv Sci (Weinh). 2022; 9:e2201409. 10.1002/advs.202201409 35822667 PMC9443461

[68] Zhang G, Li J, Purkayastha S, Tang Y, Zhang H, Yin Y, Li B, Liu G, Cai D. Hypothalamic programming of systemic ageing involving IKK-β, NF-κB and GnRH. Nature. 2013; 497:211–6. 10.1038/nature12143 23636330 PMC3756938

[69] Pyo JO, Yoo SM, Ahn HH, Nah J, Hong SH, Kam TI, Jung S, Jung YK. Overexpression of Atg5 in mice activates autophagy and extends lifespan. Nat Commun. 2013; 4:2300. 10.1038/ncomms3300 23939249 PMC3753544

[70] Liu X, Jiang N, Hughes B, Bigras E, Shoubridge E, Hekimi S. Evolutionary conservation of the clk-1-dependent mechanism of longevity: loss of mclk1 increases cellular fitness and lifespan in mice. Genes Dev. 2005; 19:2424–34. 10.1101/gad.1352905 16195414 PMC1257397

[71] Zhang Y, Xie Y, Berglund ED, Coate KC, He TT, Katafuchi T, Xiao G, Potthoff MJ, Wei W, Wan Y, Yu RT, Evans RM, Kliewer SA, Mangelsdorf DJ. The starvation hormone, fibroblast growth factor-21, extends lifespan in mice. Elife. 2012; 1:e00065. 10.7554/eLife.00065 23066506 PMC3466591

[72] Satoh A, Brace CS, Rensing N, Cliften P, Wozniak DF, Herzog ED, Yamada KA, Imai S. Sirt1 extends life span and delays aging in mice through the regulation of Nk2 homeobox 1 in the DMH and LH. Cell Metab. 2013; 18:416–30. 10.1016/j.cmet.2013.07.013 24011076 PMC3794712

[73] Harper JM, Wilkinson JE, Miller RA. Macrophage migration inhibitory factor-knockout mice are long lived and respond to caloric restriction. FASEB J. 2010; 24:2436–42. 10.1096/fj.09-152223 20219983 PMC2887269

[74] Ribeiro R, Macedo JC, Costa M, Ustiyan V, Shindyapina AV, Tyshkovskiy A, Gomes RN, Castro JP, Kalin TV, Vasques-Nóvoa F, Nascimento DS, Dmitriev SE, Gladyshev VN, et al. In vivo cyclic induction of the FOXM1 transcription factor delays natural and progeroid aging phenotypes and extends healthspan. Nat Aging. 2022; 2:397–411. 10.1038/s43587-022-00209-9 37118067

[75] Harrison DE, Strong R, Allison DB, Ames BN, Astle CM, Atamna H, Fernandez E, Flurkey K, Javors MA, Nadon NL, Nelson JF, Pletcher S, Simpkins JW, et al. Acarbose, 17-α-estradiol, and nordihydroguaiaretic acid extend mouse lifespan preferentially in males. Aging Cell. 2014; 13:273–82. 10.1111/acel.12170 24245565 PMC3954939

[76] Harrison DE, Strong R, Alavez S, Astle CM, DiGiovanni J, Fernandez E, Flurkey K, Garratt M, Gelfond JAL, Javors MA, Levi M, Lithgow GJ, Macchiarini F, et al. Acarbose improves health and lifespan in aging HET3 mice. Aging Cell. 2019; 18:e12898. 10.1111/acel.12898 30688027 PMC6413665

[77] Smith BJ, Miller RA, Ericsson AC, Harrison DC, Strong R, Schmidt TM. Changes in the gut microbiome and fermentation products concurrent with enhanced longevity in acarbose-treated mice. BMC Microbiol. 2019; 19:130. 10.1186/s12866-019-1494-7 31195972 PMC6567620

[78] Filfan M, Olaru A, Udristoiu I, Margaritescu C, Petcu E, Hermann DM, Popa-Wagner A. Long-term treatment with spermidine increases health span of middle-aged Sprague-Dawley male rats. Geroscience. 2020; 42:937–49. 10.1007/s11357-020-00173-5 32285289 PMC7287009

[79] Eisenberg T, Abdellatif M, Schroeder S, Primessnig U, Stekovic S, Pendl T, Harger A, Schipke J, Zimmermann A, Schmidt A, Tong M, Ruckenstuhl C, Dammbrueck C, et al. Cardioprotection and lifespan extension by the natural polyamine spermidine. Nat Med. 2016; 22:1428–38. 10.1038/nm.4222 27841876 PMC5806691

[80] Singh P, Gollapalli K, Mangiola S, Schranner D, Yusuf MA, Chamoli M, Shi SL, Lopes Bastos B, Nair T, Riermeier A, Vayndorf EM, Wu JZ, Nilakhe A, et al. Taurine deficiency as a driver of aging. Science. 2023; 380:eabn9257. 10.1126/science.abn9257 37289866 PMC10630957

[81] Miller RA, Harrison DE, Astle CM, Bogue MA, Brind J, Fernandez E, Flurkey K, Javors M, Ladiges W, Leeuwenburgh C, Macchiarini F, Nelson J, Ryazanov AG, et al. Glycine supplementation extends lifespan of male and female mice. Aging Cell. 2019; 18:e12953. 10.1111/acel.12953 30916479 PMC6516426

[82] Davidsohn N, Pezone M, Vernet A, Graveline A, Oliver D, Slomovic S, Punthambaker S, Sun X, Liao R, Bonventre JV, Church GM. A single combination gene therapy treats multiple age-related diseases. Proc Natl Acad Sci U S A. 2019; 116:23505–11. 10.1073/pnas.1910073116 31685628 PMC6876218

[83] Blagosklonny MV. Once again on rapamycin-induced insulin resistance and longevity: despite of or owing to. Aging (Albany NY). 2012; 4:350–8. 10.18632/aging.100461 22683661 PMC3384435

[84] Schindler CE, Partap U, Patchen BK, Swoap SJ. Chronic rapamycin treatment causes diabetes in male mice. Am J Physiol Regul Integr Comp Physiol. 2014; 307:R434–43. 10.1152/ajpregu.00123.2014 24965794 PMC4137151

[85] Cheng CJ, Gelfond JAL, Strong R, Nelson JF. Genetically heterogeneous mice exhibit a female survival advantage that is age-and site-specific: Results from a large multi-site study. Aging Cell. 2019; 18:e12905. 10.1111/acel.12905 30801953 PMC6516160

[86] Jiang Z, Wang J, Imai D, Snider T, Klug J, Mangalindan R, Morton J, Zhu L, Salmon AB, Wezeman J, Hu J, Menon V, Marka N, et al. Short term treatment with a cocktail of rapamycin, acarbose and phenylbutyrate delays aging phenotypes in mice. Sci Rep. 2022; 12:7300. 10.1038/s41598-022-11229-1 35508491 PMC9067553

[87] Wiley JC, Pettan-Brewer C, Ladiges WC. Phenylbutyric acid reduces amyloid plaques and rescues cognitive behavior in AD transgenic mice. Aging Cell. 2011; 10:418–28. 10.1111/j.1474-9726.2011.00680.x 21272191

[88] Crossland H, Smith K, Idris I, Phillips BE, Atherton PJ, Wilkinson DJ. Phenylbutyrate, a branched-chain amino acid keto dehydrogenase activator, promotes branched-chain amino acid metabolism and induces muscle catabolism in C2C12 cells. Exp Physiol. 2021; 106:585–92. 10.1113/EP089223 33369803 PMC9291829

[89] Spindler SR, Mote PL, Flegal JM. Combined statin and angiotensin-converting enzyme (ACE) inhibitor treatment increases the lifespan of long-lived F1 male mice. Age (Dordr). 2016; 38:379–91. 10.1007/s11357-016-9948-4 27590905 PMC5266223

[90] Strong R, Miller RA, Cheng CJ, Nelson JF, Gelfond J, Allani SK, Diaz V, Dorigatti AO, Dorigatti J, Fernandez E, Galecki A, Ginsburg B, Hamilton KL, et al. Lifespan benefits for the combination of rapamycin plus acarbose and for captopril in genetically heterogeneous mice. Aging Cell. 2022; 21:e13724. 10.1111/acel.13724 36179270 PMC9741502

[91] Kumar P, Osahon OW, Sekhar RV. GlyNAC (Glycine and N-Acetylcysteine) Supplementation in Mice Increases Length of Life by Correcting Glutathione Deficiency, Oxidative Stress, Mitochondrial Dysfunction, Abnormalities in Mitophagy and Nutrient Sensing, and Genomic Damage. Nutrients. 2022; 14:1114. 10.3390/nu14051114 35268089 PMC8912885

[92] Zhang B, Gladyshev VN. How can aging be reversed? Exploring rejuvenation from a damage-based perspective. Adv Genet (Hoboken). 2020; 1:e10025. 10.1002/ggn2.10025 36619246 PMC9744548

[93] Matheu A, Maraver A, Klatt P, Flores I, Garcia-Cao I, Borras C, Flores JM, Viña J, Blasco MA, Serrano M. Delayed ageing through damage protection by the Arf/p53 pathway. Nature. 2007; 448:375–9. 10.1038/nature05949 17637672

[94] Schriner SE, Linford NJ, Martin GM, Treuting P, Ogburn CE, Emond M, Coskun PE, Ladiges W, Wolf N, Van Remmen H, Wallace DC, Rabinovitch PS. Extension of murine life span by overexpression of catalase targeted to mitochondria. Science. 2005; 308:1909–11. 10.1126/science.1106653 15879174

[95] Macip CC, Hasan R, Hoznek V, Kim J, Lu YR, Metzger LE 4th, Sethna S, Davidsohn N. Gene Therapy-Mediated Partial Reprogramming Extends Lifespan and Reverses Age-Related Changes in Aged Mice. Cell Reprogram. 2024; 26:24–32. 10.1089/cell.2023.0072 38381405 PMC10909732

[96] Bartke A, Wright JC, Mattison JA, Ingram DK, Miller RA, Roth GS. Extending the lifespan of long-lived mice. Nature. 2001; 414:412. 10.1038/35106646 11719795

[97] Sun LY, Spong A, Swindell WR, Fang Y, Hill C, Huber JA, Boehm JD, Westbrook R, Salvatori R, Bartke A. Growth hormone-releasing hormone disruption extends lifespan and regulates response to caloric restriction in mice. Elife. 2013; 2:e01098. 10.7554/eLife.01098 24175087 PMC3810783

[98] Masternak MM, Panici JA, Bonkowski MS, Hughes LF, Bartke A. Insulin sensitivity as a key mediator of growth hormone actions on longevity. J Gerontol A Biol Sci Med Sci. 2009; 64:516–21. 10.1093/gerona/glp024 19304940 PMC2667133

[99] Bonkowski MS, Rocha JS, Masternak MM, Al Regaiey KA, Bartke A. Targeted disruption of growth hormone receptor interferes with the beneficial actions of calorie restriction. Proc Natl Acad Sci U S A. 2006; 103:7901–5. 10.1073/pnas.0600161103 16682650 PMC1458512

[100] Zha Y, Taguchi T, Nazneen A, Shimokawa I, Higami Y, Razzaque MS. Genetic suppression of GH-IGF-1 activity, combined with lifelong caloric restriction, prevents age-related renal damage and prolongs the life span in rats. Am J Nephrol. 2008; 28:755–64. 10.1159/000128607 18434714

[101] Linz W, Jessen T, Becker RH, Schölkens BA, Wiemer G. Long-term ACE inhibition doubles lifespan of hypertensive rats. Circulation. 1997; 96:3164–72. 10.1161/01.cir.96.9.3164 9386189

[102] Getz GS, Reardon CA. Animal models of atherosclerosis. Arterioscler Thromb Vasc Biol. 2012; 32:1104–15. 10.1161/ATVBAHA.111.237693 22383700 PMC3331926

[103] Geng L, Liu Z, Wang S, Sun S, Ma S, Liu X, Chan P, Sun L, Song M, Zhang W, Liu GH, Qu J. Low-dose quercetin positively regulates mouse healthspan. Protein Cell. 2019; 10:770–5. 10.1007/s13238-019-0646-8 31325157 PMC6776572

[104] Xu M, Pirtskhalava T, Farr JN, Weigand BM, Palmer AK, Weivoda MM, Inman CL, Ogrodnik MB, Hachfeld CM, Fraser DG, Onken JL, Johnson KO, Verzosa GC, et al. Senolytics improve physical function and increase lifespan in old age. Nat Med. 2018; 24:1246–56. 10.1038/s41591-018-0092-9 29988130 PMC6082705

[105] Novais EJ, Tran VA, Johnston SN, Darris KR, Roupas AJ, Sessions GA, Shapiro IM, Diekman BO, Risbud MV. Long-term treatment with senolytic drugs Dasatinib and Quercetin ameliorates age-dependent intervertebral disc degeneration in mice. Nat Commun. 2021; 12:5213. 10.1038/s41467-021-25453-2 34480023 PMC8417260

[106] Cavalcante MB, Saccon TD, Nunes ADC, Kirkland JL, Tchkonia T, Schneider A, Masternak MM. Dasatinib plus quercetin prevents uterine age-related dysfunction and fibrosis in mice. Aging (Albany NY). 2020; 12:2711–22. 10.18632/aging.102772 31955151 PMC7041753

[107] Kirkland JL, Tchkonia T. Senolytic drugs: from discovery to translation. J Intern Med. 2020; 288:518–36. 10.1111/joim.13141 32686219 PMC7405395

[108] Hickson LJ, Langhi Prata LGP, Bobart SA, Evans TK, Giorgadze N, Hashmi SK, Herrmann SM, Jensen MD, Jia Q, Jordan KL, Kellogg TA, Khosla S, Koerber DM, et al. Senolytics decrease senescent cells in humans: Preliminary report from a clinical trial of Dasatinib plus Quercetin in individuals with diabetic kidney disease. EBioMedicine. 2019; 47:446–56. 10.1016/j.ebiom.2019.08.069 31542391 PMC6796530

[109] Justice JN, Nambiar AM, Tchkonia T, LeBrasseur NK, Pascual R, Hashmi SK, Prata L, Masternak MM, Kritchevsky SB, Musi N, Kirkland JL. Senolytics in idiopathic pulmonary fibrosis: Results from a first-in-human, open-label, pilot study. EBioMedicine. 2019; 40:554–63. 10.1016/j.ebiom.2018.12.052 30616998 PMC6412088

[110] Kirkland JL, Tchkonia T, Zhu Y, Niedernhofer LJ, Robbins PD. The Clinical Potential of Senolytic Drugs. J Am Geriatr Soc. 2017; 65:2297–301. 10.1111/jgs.14969 28869295 PMC5641223

[111] La Rosée P, Martiat P, Leitner A, Klag T, Müller MC, Erben P, Schenk T, Saussele S, Hochhaus A. Improved tolerability by a modified intermittent treatment schedule of dasatinib for patients with chronic myeloid leukemia resistant or intolerant to imatinib. Ann Hematol. 2013; 92:1345–50. 10.1007/s00277-013-1769-2 23625298

[112] Bai Z, Yang P, Yu F, Li Z, Yao Z, Martinez J, Li M, Xu H. Combining adoptive NK cell infusion with a dopamine-releasing peptide reduces senescent cells in aged mice. Cell Death Dis. 2022; 13:305. 10.1038/s41419-022-04562-w 35383143 PMC8983684

[113] Pifferi F, Terrien J, Marchal J, Dal-Pan A, Djelti F, Hardy I, Chahory S, Cordonnier N, Desquilbet L, Hurion M, Zahariev A, Chery I, Zizzari P, et al. Caloric restriction increases lifespan but affects brain integrity in grey mouse lemur primates. Commun Biol. 2018; 1:30. 10.1038/s42003-018-0024-8 30271916 PMC6123706

[114] Shimokawa I, Komatsu T, Hayashi N, Kim SE, Kawata T, Park S, Hayashi H, Yamaza H, Chiba T, Mori R. The life-extending effect of dietary restriction requires Foxo3 in mice. Aging Cell. 2015; 14:707–9. 10.1111/acel.12340 25808402 PMC4531086

[115] Ma L, Dong W, Wang R, Li Y, Xu B, Zhang J, Zhao Z, Wang Y. Effect of caloric restriction on the SIRT1/mTOR signaling pathways in senile mice. Brain Res Bull. 2015; 116:67–72. 10.1016/j.brainresbull.2015.06.004 26135885

[116] Chen D, Bruno J, Easlon E, Lin SJ, Cheng HL, Alt FW, Guarente L. Tissue-specific regulation of SIRT1 by calorie restriction. Genes Dev. 2008; 22:1753–7. 10.1101/gad.1650608 18550784 PMC2492662

[117] Arum O, Bonkowski MS, Rocha JS, Bartke A. The growth hormone receptor gene-disrupted mouse fails to respond to an intermittent fasting diet. Aging Cell. 2009; 8:756–60. 10.1111/j.1474-9726.2009.00520.x 19747233 PMC2783987

[118] Takahashi K, Yamanaka S. Induction of pluripotent stem cells from mouse embryonic and adult fibroblast cultures by defined factors. Cell. 2006; 126:663–76. 10.1016/j.cell.2006.07.024 16904174

[119] Takahashi K, Tanabe K, Ohnuki M, Narita M, Ichisaka T, Tomoda K, Yamanaka S. Induction of pluripotent stem cells from adult human fibroblasts by defined factors. Cell. 2007; 131:861–72. 10.1016/j.cell.2007.11.019 18035408

[120] Simpson DJ, Olova NN, Chandra T. Cellular reprogramming and epigenetic rejuvenation. Clin Epigenetics. 2021; 13:170. 10.1186/s13148-021-01158-7 34488874 PMC8419998

[121] Browder KC, Reddy P, Yamamoto M, Haghani A, Guillen IG, Sahu S, Wang C, Luque Y, Prieto J, Shi L, Shojima K, Hishida T, Lai Z, et al. In vivo partial reprogramming alters age-associated molecular changes during physiological aging in mice. Nat Aging. 2022; 2:243–53. 10.1038/s43587-022-00183-2 37118377

[122] Sadowska-Bartosz I, Bartosz G. Effect of antioxidants supplementation on aging and longevity. Biomed Res Int. 2014; 2014:404680. 10.1155/2014/404680 24783202 PMC3982418

[123] Bjelakovic G, Nikolova D, Gluud C. Antioxidant supplements and mortality. Curr Opin Clin Nutr Metab Care. 2014; 17:40–4. 10.1097/MCO.0000000000000009 24241129

[124] Pérez VI, Van Remmen H, Bokov A, Epstein CJ, Vijg J, Richardson A. The overexpression of major antioxidant enzymes does not extend the lifespan of mice. Aging Cell. 2009; 8:73–5. 10.1111/j.1474-9726.2008.00449.x 19077044 PMC2667893

[125] Sun J, Molitor J, Tower J. Effects of simultaneous over-expression of Cu/ZnSOD and MnSOD on Drosophila melanogaster life span. Mech Ageing Dev. 2004; 125:341–9. 10.1016/j.mad.2004.01.009 15130751

[126] Pal K, Raghuram GV, Dsouza J, Shinde S, Jadhav V, Shaikh A, Rane B, Tandel H, Kondhalkar D, Chaudhary S, Mittra I. A pro-oxidant combination of resveratrol and copper down-regulates multiple biological hallmarks of ageing and neurodegeneration in mice. Sci Rep. 2022; 12:17209. 10.1038/s41598-022-21388-w 36241685 PMC9568542

[127] Flurkey K, Astle CM, Harrison DE. Life extension by diet restriction and N-acetyl-L-cysteine in genetically heterogeneous mice. J Gerontol A Biol Sci Med Sci. 2010; 65:1275–84. 10.1093/gerona/glq155 20819793 PMC2990268

[128] Spindler SR, Mote PL, Flegal JM, Teter B. Influence on longevity of blueberry, cinnamon, green and black tea, pomegranate, sesame, curcumin, morin, pycnogenol, quercetin, and taxifolin fed iso-calorically to long-lived, F1 hybrid mice. Rejuvenation Res. 2013; 16:143–51. 10.1089/rej.2012.1386 23432089

[129] Gesing A, Wiesenborn D, Do A, Menon V, Schneider A, Victoria B, Stout MB, Kopchick JJ, Bartke A, Masternak MM. A Long-lived Mouse Lacking Both Growth Hormone and Growth Hormone Receptor: A New Animal Model for Aging Studies. J Gerontol A Biol Sci Med Sci. 2017; 72:1054–61. 10.1093/gerona/glw193 27688483 PMC5861925

[130] Zhou JX, Dhawan S, Fu H, Snyder E, Bottino R, Kundu S, Kim SK, Bhushan A. Combined modulation of polycomb and trithorax genes rejuvenates β cell replication. J Clin Invest. 2013; 123:4849–58. 10.1172/JCI69468 24216481 PMC3809789

[131] Fahy GM, Brooke RT, Watson JP, Good Z, Vasanawala SS, Maecker H, Leipold MD, Lin DTS, Kobor MS, Horvath S. Reversal of epigenetic aging and immunosenescent trends in humans. Aging Cell. 2019; 18:e13028. 10.1111/acel.13028 31496122 PMC6826138

[132] Lu Y, Brommer B, Tian X, Krishnan A, Meer M, Wang C, Vera DL, Zeng Q, Yu D, Bonkowski MS, Yang JH, Zhou S, Hoffmann EM, et al. Reprogramming to recover youthful epigenetic information and restore vision. Nature. 2020; 588:124–9. 10.1038/s41586-020-2975-4 33268865 PMC7752134

[133] Liao CY, Kennedy BK. Mouse models and aging: longevity and progeria. Curr Top Dev Biol. 2014; 109:249–85. 10.1016/B978-0-12-397920-9.00003-2 24947239

[134] Villa-Bellosta R. Synthesis of Extracellular Pyrophosphate Increases in Vascular Smooth Muscle Cells During Phosphate-Induced Calcification. Arterioscler Thromb Vasc Biol. 2018; 38:2137–47. 10.1161/ATVBAHA.118.311444 30002059

[135] Villa-Bellosta R. ATP-based therapy prevents vascular calcification and extends longevity in a mouse model of Hutchinson-Gilford progeria syndrome. Proc Natl Acad Sci U S A. 2019; 116:23698–704. 10.1073/pnas.1910972116 31690656 PMC6876227

[136] Varela I, Pereira S, Ugalde AP, Navarro CL, Suárez MF, Cau P, Cadiñanos J, Osorio FG, Foray N, Cobo J, de Carlos F, Lévy N, Freije JM, López-Otín C. Combined treatment with statins and aminobisphosphonates extends longevity in a mouse model of human premature aging. Nat Med. 2008; 14:767–72. 10.1038/nm1786 18587406

[137] Ocampo A, Reddy P, Martinez-Redondo P, Platero-Luengo A, Hatanaka F, Hishida T, Li M, Lam D, Kurita M, Beyret E, Araoka T, Vazquez-Ferrer E, Donoso D, et al. In Vivo Amelioration of Age-Associated Hallmarks by Partial Reprogramming. Cell. 2016; 167:1719–33.e12. 10.1016/j.cell.2016.11.052 27984723 PMC5679279

[138] Jolly CA, Muthukumar A, Avula CP, Troyer D, Fernandes G. Life span is prolonged in food-restricted autoimmune-prone (NZB x NZW)F(1) mice fed a diet enriched with (n-3) fatty acids. J Nutr. 2001; 131:2753–60. 10.1093/jn/131.10.2753 11584100

[139] Walker SE, Anver MR. Stimulated autoantibody response and increased longevity in NZB/NZW mice treated with cyclophosphamide and tilorone. Clin Exp Immunol. 1978; 33:453–62. 737897 PMC1537448

[140] Hong MK, Han Y, Park HJ, Shin MR, Roh SS, Kwon EY. The Synergistic Action of Metformin and *Glycyrrhiza uralensis* Fischer Extract Alleviates Metabolic Disorders in Mice with Diet-Induced Obesity. Int J Mol Sci. 2023; 24:936. 10.3390/ijms24020936 36674447 PMC9862386

[141] Islam MT, Tuday E, Allen S, Kim J, Trott DW, Holland WL, Donato AJ, Lesniewski LA. Senolytic drugs, dasatinib and quercetin, attenuate adipose tissue inflammation, and ameliorate metabolic function in old age. Aging Cell. 2023; 22:e13767. 10.1111/acel.13767 36637079 PMC9924942

[142] Kumar P, Liu C, Hsu JW, Chacko S, Minard C, Jahoor F, Sekhar RV. Glycine and N-acetylcysteine (GlyNAC) supplementation in older adults improves glutathione deficiency, oxidative stress, mitochondrial dysfunction, inflammation, insulin resistance, endothelial dysfunction, genotoxicity, muscle strength, and cognition: Results of a pilot clinical trial. Clin Transl Med. 2021; 11:e372. 10.1002/ctm2.372 33783984 PMC8002905

[143] Lewis CJ, de Grey AD. Combining rejuvenation interventions in rodents: a milestone in biomedical gerontology whose time has come. Expert Opin Ther Targets. 2024; 28:501–11. 10.1080/14728222.2024.2330425 38477630

[144] Most J, Tosti V, Redman LM, Fontana L. Calorie restriction in humans: An update. Ageing Res Rev. 2017; 39:36–45. 10.1016/j.arr.2016.08.005 27544442 PMC5315691

[145] Shchaslyvyi AY, Antonenko SV, Tesliuk MG, Telegeev GD. Current State of Human Gene Therapy: Approved Products and Vectors. Pharmaceuticals (Basel). 2023; 16:1416. 10.3390/ph16101416 37895887 PMC10609992

[146] Rafikova E, Nemirovich-Danchenko N, Ogmen A, Parfenenkova A, Velikanova A, Tikhonov S, Peshkin L, Rafikov K, Spiridonova O, Belova Y, Glinin T, Egorova A, Batin M. Open Genes-a new comprehensive database of human genes associated with aging and longevity. Nucleic Acids Res. 2024; 52:D950–62. 10.1093/nar/gkad712 37665017 PMC10768108

[147] Brooks WW, Conrad CH. Myocardial fibrosis in transforming growth factor beta(1)heterozygous mice. J Mol Cell Cardiol. 2000; 32:187–95. 10.1006/jmcc.1999.1065 10722796

[148] Redondo S, Navarro-Dorado J, Ramajo M, Medina Ú, Tejerina T. The complex regulation of TGF-β in cardiovascular disease. Vasc Health Risk Manag. 2012; 8:533–9. 10.2147/VHRM.S28041 23028232 PMC3446857

[149] Willcox BJ, Donlon TA, He Q, Chen R, Grove JS, Yano K, Masaki KH, Willcox DC, Rodriguez B, Curb JD. FOXO3A genotype is strongly associated with human longevity. Proc Natl Acad Sci U S A. 2008; 105:13987–92. 10.1073/pnas.0801030105 18765803 PMC2544566

[150] Li N, Luo H, Liu X, Ma S, Lin H, Chen R, Hao F, Zhang D. Association study of polymorphisms in FOXO3, AKT1 and IGF-2R genes with human longevity in a Han Chinese population. Oncotarget. 2016; 7:23–32. 10.18632/oncotarget.6627 26683100 PMC4807980

[151] Ji JS, Liu L, Shu C, Yan LL, Zeng Y. Sex Difference and Interaction of SIRT1 and FOXO3 Candidate Longevity Genes on Life Expectancy: A 10-Year Prospective Longitudinal Cohort Study. J Gerontol A Biol Sci Med Sci. 2022; 77:1557–63. 10.1093/gerona/glab378 34928346 PMC9373943

[152] Chen R, Morris BJ, Donlon TA, Masaki KH, Willcox DC, Davy PMC, Allsopp RC, Willcox BJ. *FOXO3* longevity genotype mitigates the increased mortality risk in men with a cardiometabolic disease. Aging (Albany NY). 2020; 12:23509–24. 10.18632/aging.202175 33260156 PMC7762472

[153] Zhao Y, Liu YS. Longevity Factor FOXO3: A Key Regulator in Aging-Related Vascular Diseases. Front Cardiovasc Med. 2021; 8:778674. 10.3389/fcvm.2021.778674 35004893 PMC8733402

[154] Boehm AM, Khalturin K, Anton-Erxleben F, Hemmrich G, Klostermeier UC, Lopez-Quintero JA, Oberg HH, Puchert M, Rosenstiel P, Wittlieb J, Bosch TC. FoxO is a critical regulator of stem cell maintenance in immortal Hydra. Proc Natl Acad Sci U S A. 2012; 109:19697–702. 10.1073/pnas.1209714109 23150562 PMC3511741

[155] Boehm AM, Rosenstiel P, Bosch TC. Stem cells and aging from a quasi-immortal point of view. Bioessays. 2013; 35:994–1003. 10.1002/bies.201300075 24037777

[156] Libina N, Berman JR, Kenyon C. Tissue-specific activities of C. elegans DAF-16 in the regulation of lifespan. Cell. 2003; 115:489–502. 10.1016/s0092-8674(03)00889-4 14622602

[157] Mulvey L, Sinclair A, Selman C. Lifespan modulation in mice and the confounding effects of genetic background. J Genet Genomics. 2014; 41:497–503. 10.1016/j.jgg.2014.06.002 25269675 PMC4257991

[158] Selman C, Swindell WR. Putting a strain on diversity. EMBO J. 2018; 37:e100862. 10.15252/embj.2018100862 30389663 PMC6236330

